# Optogenetic Strategies for Optimizing the Performance of Phospholipids Biosensors

**DOI:** 10.1002/advs.202403026

**Published:** 2024-07-29

**Authors:** Yuanfa Yao, Xiayan Lou, Luhong Jin, Weiyun Sun, Jingfang Liu, Yunyue Chen, Sunying Cheng, Tengjiao Zhao, Shuwei Ke, Luhao Zhang, Yingke Xu, Lian He, Hanbing Li

**Affiliations:** ^1^ Institute of Pharmacology College of Pharmaceutical Science Zhejiang University of Technology Hangzhou 310014 China; ^2^ School of Information Science and Technology Hangzhou Normal University Hangzhou Zhejiang 311121 China; ^3^ Department of Biomedical Engineering Key Laboratory of Biomedical Engineering of Ministry of Education State Key Laboratory of Extreme Photonics and Instrumentation Zhejiang Provincial Key Laboratory of Cardio‐Cerebral Vascular Detection Technology and Medicinal Effectiveness Appraisal Zhejiang University Hangzhou 310027 China; ^4^ Department of Endocrinology Children's Hospital of Zhejiang University School of Medicine National Clinical Research Center for Children's Health Hangzhou Zhejiang 310051 China; ^5^ Department of Pharmacology Joint Laboratory of Guangdong‐Hong Kong Universities for Vascular Homeostasis and Diseases School of Medicine Southern University of Science and Technology Shenzhen 518055 China

**Keywords:** optogentics, phase separation, phosphatidic acid, phospholipase D, phospholipid biosensors

## Abstract

High‐performance biosensors play a crucial role in elucidating the intricate spatiotemporal regulatory roles and dynamics of membrane phospholipids. However, enhancing the sensitivity and imaging performance remains a significant challenge. Here, optogenetic‐based strategies are presented to optimize phospholipid biosensors. These strategies involves presequestering unbound biosensors in the cell nucleus and regulating their cytosolic levels with blue light to minimize background signal interference in phospholipid detection, particularly under conditions of high expression levels of biosensor. Furthermore, optically controlled phase separation and the SunTag system are employed to generate punctate probes for substrate detection, thereby amplifying biosensor signals and enhancing visualization of the detection process. These improved phospholipid biosensors hold great potential for enhancing the understanding of the spatiotemporal dynamics and regulatory roles of membrane lipids in live cells and the methodological insights in this study might be valuable for developing other high‐performance biosensors.

## Graphical abstract

This research employs an optogenetic tool to regulate the nucleocytoplasmic transport of phospholipid biosensors with light to improve their signal‐to‐background ratio (SBR). By integrating optogenetic platforms and phase separation techniques, the formation of punctate phospholipid biosensors is controlled to amplify their signal. These improved biosensors enable the investigation of the spatiotemporal dynamics and regulatory roles of membrane lipids in living cells.

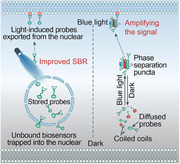

## 1 Introduction

The dynamic metabolism of membrane lipids is a rapid and intricate process that demands high‐performance probes for real‐time monitoring within living cells. Biosensors play a crucial role in unraveling the spatiotemporal regulatory functions of membrane lipids and have been widely employed to track their fluctuations in cells. A primary optimization strategy for enhancing these biosensors involves selecting sensitive phospholipid‐binding motifs from a variety of proteins of interest (POI). However, under certain conditions, such as detecting low‐abundance phospholipids, the unbound fraction resulting from the intracellular biosensor overexpression can exert varying degrees of influence on detection performance. In contrast to those efforts aimed at enhancing biosensor sensitivity, there has been relatively rare exploration into reducing background signals from the unbound biosensors to improve their signal‐to‐background ratio (SBR) or amplifying these signals during the imaging process.

To eliminate the impact of unbound biosensors on the detection of membrane phospholipids and achieve a clearer visualization of their distribution, a more effective strategy is to segregate dissociated probes to the specific region and then improve the SBR in imaging process. For example, the unbound probes can be sequestered to the nucleus through optogenetic tools, a technique platform has found extensive application in manipulating the spatiotemporal localization^[^ ^1^, ^2^, ^3^, ^4^ ^]^ and conformational dynamics of POIs,^[^ ^5^ ^]^ and regulating gene transcription,^[^ ^6^, ^7^, ^8^ ^]^ modulating ion channel states,^[^ ^9^ ^]^ and facilitating the intracellular movement of organelles^[^ ^10^, ^11^ ^]^ in previous studies. The Light‐oxygen‐voltage (LOV) domain 2 (LOV2)‐based nucleocytoplasmic transport system,^[^ ^12^, ^13^ ^]^ easily activated by blue light, might serve as an ideal tool for controlling the expression levels of biosensors in the cytosol according to experimental requirements, thereby minimizing the impact of unbound biosensors on phospholipid detection. Another strategy is to amplify the signal of biosensors. the SunTag system and similar tools has been extensively used to amplify the single‐molecular signal through incorporation of a dozen of tandem short peptides, such as the basic region leucine zipper of the eukaryotic transcriptional activator protein (GCN4),^[^ ^14^, ^15^, ^16^ ^]^ hemagglutinin (HA),^[^ ^17^ ^]^ and the human immunodeficiency virus (HIV)‐1 envelope glycoprotein (gp41) peptide,^[^ ^18^ ^]^ into POIs. These peptides are then recognized by a multitude of fluorescent antibodies, resulting in the formation of punctum‐like structures that are notably brighter than the background, thus amplifying the signal. Importantly, this process requires controlling the expression levels of the POI through doxycycline (DOX) induction to prevent the influence of excessive punctum‐like structures on imaging performance. Recently, the homo‐oligomeric tags (HOTags)‐mediated phase separation has been shown to generate similar structures in cells and has been utilized to report the cellular activities.^[^ ^19^, ^20^, ^21^ ^]^ The formation of these puncta is dependent on the interactions of POIs that fused with HOTags peptides. The brightness of them is comparative to that of the SunTag system.

It remains unclear whether the aforementioned advanced techniques can enhance the performance of phospholipids biosensors. In pursuit of addressing this question, our study employed cpLOV2‐mediated nucleocytoplasmic transport to reconstruct phospholipid biosensors. This approach involves in the sequestration of unbound biosensors within the cell nucleus under dark conditions via nuclear localization sequence (NLS)‐mediated nuclear localization, which leads to a significant reduction in background noise and an improvement in SBR of the biosensors. Upon subsequent exposure to light, these stored probes were released from the nucleus, ready to bind to newly‐synthesized phospholipids in response to agonists. We applied these strategies to enhance the performance of phosphatidic acid (PA) biosensor, which showed more pronounced improvements under conditions that the biosensor has a high expression levels. Moreover, to further improve biosensors, we fused PA and phosphatidylinositol‐4‐phosphate (PI4P) binding motifs with the N‐terminal of cryptochrome‐interacting basic‐helix‐loop‐helix 1 (CIBN), respectively. By virtue of the light‐responsive interactions between Cryptochrome 2 (CRY2) and CIBN, we triggered the phase separation of their fused peptides HOTag3 and HOTag6^[^ ^19^, ^20^, ^21^ ^]^ with blue light, resulting in the formation of abundant punctum‐like structures. This novel approach not only amplified the signal of the PI4P and PA biosensors, but also rapidly assembled a cluster of biosensors with multiple copies of phospholipid‐binding motifs and enhanced their sensitivity to corresponding phospholipids, which differs from the common approaches that increase the number of phospholipid‐binding motif copies during biosensors design. Similar punctate biosensors were also achieved through the SunTag system. These punctum‐like biosensors facilitated the visualization of intracellular phospholipids, enabling the observation of low‐abundance phospholipids on cellular organelles and detection of minor concentration changes. Collectively, these refined biosensors may serve as useful tools for investigating the spatiotemporal functions of PI4P and PA. The methodological improvements proposed in this study can also serve as valuable references for the design of high‐performance biosensors.

## 2 Results and Discussion

### 2.1 Designing Optogenetics‐Based Biosensors with Circularly Permuted LOV2 (cpLOV2) to Minimize the Interference from Unbound Biosensors

In previous reports and reviews, we highlighted the significant roles of PLDs and PA in cells,^[^ ^22^, ^23^, ^24^ ^]^ especially in cancer cells. However, to gain a comprehensive understanding of their spatiotemporal regulatory roles, we need to monitor their real‐time activity and dynamics. Thus, advanced detection methods are indispensable. Baskin's group developed a sophisticated chemical detection platform based on click chemistry for labeling PA and tracking PLD activities.^[^ ^25^ ^]^ This platform facilitated the investigation of localization‐dependent regulatory roles of PLDs^[^ ^26^ ^]^ and identification of cells with heightened PLD activities.^[^ ^27^ ^]^ Apart from this technique, the detection of other membrane lipids in live cells predominantly relies on biosensors, employing specific motifs from phospholipid‐binding proteins. However, the presence of unbound biosensors can significantly impact the imaging performance, especially for low‐affinity probes and detecting low‐abundance substrates.

**Figure 1:**
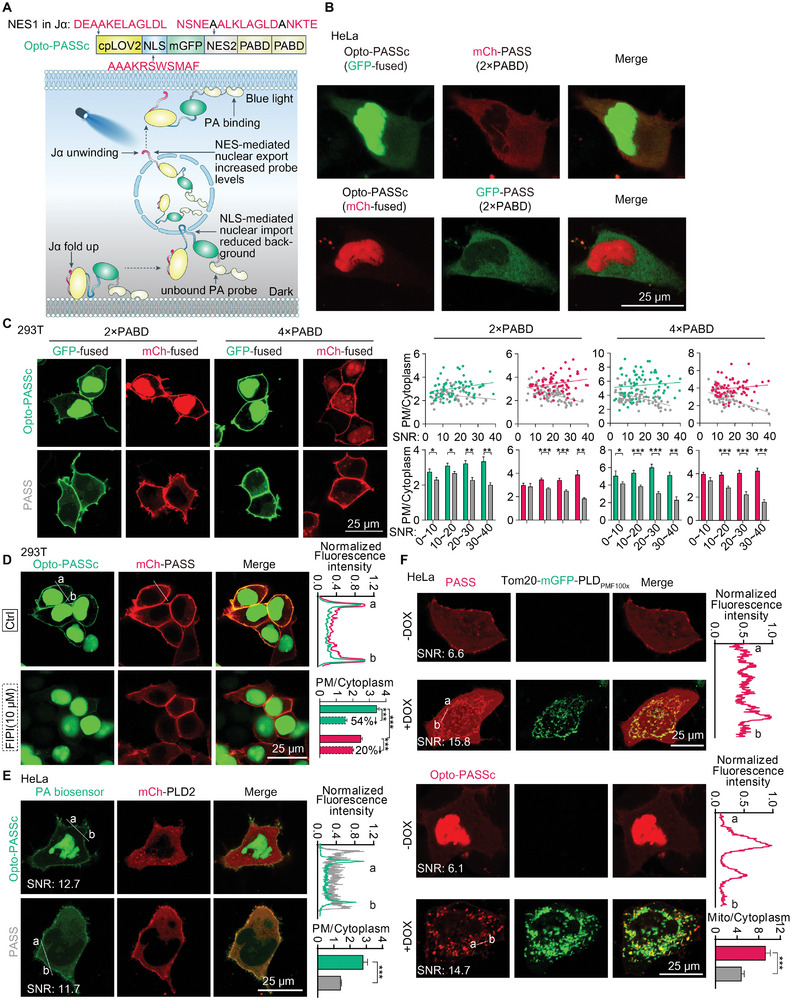
Design of optogenetics‐based phospholipid biosensor with circularly permuted LOV2 (cpLOV2). A) a schematic diagram of the cpLOV‐based strategy for improvements of membrane phospholipids biosensors, NES1 was inserted in the Jα helix of the cpLOV2 element and exposed upon blue light stimulation; two residues (leucine and isoleucine) of the NES2 were mutated into Alanine and colored in black. B) the intracellular localization of Opto‐PASSc (fused with Emerald (GFP) or mCherry (mCh)) with PASS biosensor (two copies of PABDs) in HeLa cells. C) Representative images of Opto‐PASSc and PASS with two or four copies of PABDs and the fluorescence intensity ratio of plasma membrane (PM) to the cytosol was used to assess their PA‐detecting ability in 293T cells (*n* ≥ 50 cells). SNR (the fluorescence intensity ratio of the whole cell to noise) was used to indicate the expression levels of PA biosensors. D) Opto‐PASSc and PASS were used to reflect PA levels at the PM after four‐hour treatment of PLDs inhibitor FIPI (10 × 10^−6^ m) in 293T cells (*n* ≥ 40) and E) overexpression of PLD2 in HeLa cells (*n* ≥ 10), the fluorescence intensity of white lines were normalized to the maximum and was used to reflect the SBR of the Opto‐PASSc and PASS biosensors. The PM/cytosol ratio indicated the performance of these two biosensors in detecting PA at the PM. F) Opto‐PASSc and PASS in detecting PA on the mitochondria through a tetracycline‐inducible expression of highly‐active PLD_PMF100×_, doxycycline (2 µg mL^−1^) incubation time was 5 h; the normalized fluorescence intensity of the white line (ab) was analyzed to assess the SBR of these two biosensors. SNR as indicated in the figures was used to indicate the expression levels of PA biosensors. The mito/cytosol ratio indicated the performance of these two biosensors in detecting PA at the mitochondrial (*n* ≥ 10). Data were presented as means ± SEM. Two‐tailed Student's *t*‐test is used for (C–F). The *p* values less than 0.05 were considered as significant, *p < 0.05, **p < 0.01, ***p < 0.001.

To minimize their impact, we integrated an optogenetic element to sequester unbound molecules into the nucleus. To do this, we first created a cpLOV2‐mCherry (mCh)‐NLS construct and added different nuclear export sequence 1 (NES1) at the N‐terminal Jα helix (Figure S1A, Supporting Information). Under the dark condition, the majority of these proteins were localized within the cell nucleus ascribe to the presence of NLS (Figure S1B, Supporting Information). After exposure to blue light, the N‐terminal Jα helix was supposed to unfold and expose the fused NES to mediate the nuclear export. Two out of the seven tested NES sequences exhibited the expected nuclear export and the NES (DEAAKELAGLDL) demonstrated the highest effectiveness (Figure S1A,B, Supporting Information). Subsequently, we conducted a comparative analysis of the PA binding capabilities of different PA binding domains (PABDs). Notably, the PABD from yeast overproduction of inositol 1 gene product (Opi1p) and human Raf1 did not perform as anticipated in detecting PA levels at the PM (Figure S1C,D, Supporting Information). Consequently, we selected the PABD from the PA biosensor PASS. This wildly‐used probe were developed by Du's group and used the PABD (51‐91) from SPO20 for PA‐binding.^[^ ^28^ ^]^ They inserted a NES (namely NES2 in **Figure** **1**A) to counteract the nuclear localization of PABD, thus promoting increased expression of the biosensor in the cytosol. Owing to the potential interference of this NES with the action of NLS that intended to seclude the unbound biosensors into the nucleus, we mutated two key hydrophobic residues (leucine and isoleucine) into alanine to deactivate this NES. The optimized cpLOV2 was added to the N‐terminal of the PASS biosensor and we introduced two or more tandem copies of PABD to increase the PA‐binding ability (Figure 1A). This optogenetics‐based PA biosensor was subsequently renamed as optogenetics PA super sensor based on cpLOV2 (Opto‐PASSc) (Figure 1A).

The mutation of NES2 further increased the NLS‐mediated nuclear localization of Opto‐PASSc (Figure S2A,B, Supporting Information) and these probes predominantly localized to the nucleus under basal state (Figure 1B), partially suggesting a low basal levels of PA in the cytoplasm. Additionally, they also exhibited colocalization with the referenced PA probes RFP‐PASS and GFP‐PASS in HeLa and 293T cells (Figure S2C,D, Supporting Information). Both GFP‐ and mCh‐tagged Opto‐PASSc, as well as the corresponding non‐optimized PASS biosensor, were clearly localized at the PM in 293T cells, indicating high levels of PA (Figure 1C and Figure S2E, Supporting Information). Given that the expression levels of different PA biosensor directly influenced their detecting performance, we used signal‐to‐noise ratio (SNR) (the ratio of the mean fluorescence intensity of the whole cell to the noise) to indicate their expression levels and classified them into four group according to SNR values (Figure 1C right panel). The ratio of plasma membrane (PM) fluorescence intensity to that of the cytoplasm was analyzed and it was considered as the SBR of different PA biosensors. As the expression levels of PA biosensor increased, the interference from unbound PASS probes in the cytoplasm became more pronounced, leading to a decreasing trend in their SBR values. In contrast, the optimized Opto‐PASS probes, regardless of fluorescent tags or PABD copies, were unaffected by this factor (Figure 1C, right panel). Moreover, Opto‐PASSc demonstrated a lower background signal in the cytosol, and their ratio of PM to cytoplasm was higher compared to PASS (Figure 1C, right panel). This advantage became more pronounced as the probe expression levels increased, which partially indicates an enhancement in the PA‐detecting performance of Opto‐PASSc. Additionally, our results also indicated that increasing the number of PABD copies partially improved the PA‐detecting ability of both Opto‐PASSc and PASS biosensors (Figure 1C). We also generated an Opto‐PASSc variant with two mutated PABDs that is incapable of binding PA. The results demonstrated that this construct exhibited reduced localization at the PM compared to Opto‐PASSc with wild‐type PABDs (Figure S2F, Supporting Information), partially suggesting its specificity for PA binding.

As both Opto‐PASSc and PASS exhibited high basal levels of PA at the plasma membrane in 293T cells, and considering that PLD1 and PLD2 are the main enzymes responsible for PA biosynthesis through hydrolysis of phosphatidylcholine (PC), we utilized FIPI, a dual inhibitor of PLD1 and PLD2, to inhibit their activity in PA production. Subsequently, we employed PA biosensors to evaluate the effectiveness of this pharmacological inhibition. Both these two biosensors reflected PA reduction at the PM after a four‐hour FIPI treatment, but the SBR of the Opto‐PASSc probe was lower than the control PASS probe (Figure 1D, top right panel). Quantitative analysis showed that the drop in PA levels was more obvious with the Opto‐PASSc probe (Figure 1D, bottom right panel). Additionally, the Opto‐PASSc probe also demonstrated consistent SBR improvement in cells with comparable expression levels of both biosensors after overexpression of PLD2 (Figure 1E, top right panel). Its PM/cytoplasm ratio was higher than that of the PASS biosensor (Figure 1E, bottom right panel). Given that the basal activities of endogenous PLDs were typically low, their activation requires additional stimulation,^[^ ^22^, ^23^, ^24^ ^]^ such as G‐protein‐coupled receptor agonists and protein kinase C (PKC) activation. To assess the PA‐detecting performance of our enhanced biosensor in the cytosol, we generated a tetracycline‐inducible construct of PLD with elevated basal activity (referred to here as bacterial Streptomyces sp. strain PMF PLD with a 100‐fold increase in activity (PLD_PMF100×_), this variant was called SuperPLD and was created by Baskin's group^[^ ^29^ ^]^). Its basal activity is a hundredfold higher than that of the wild‐type PLD_PMF_. After a five‐hour induction with DOX, we could clearly observe that the Tom20‐fused PLD_PMF100×_ is located on the mitochondria in contrast to the untreated group. This resulted in the production of PA on the mitochondria, which was detected by both the Opto‐PASSc and PASS biosensors (Figure 1F). We analyzed the SBR and the mito/cytoplasm fluorescence intensity ratio of these biosensors, respectively and the Opto‐PASSc displayed a higher SBR and a higher ratio in the mito/cytoplasm fluorescence intensity (Figure 1F, right panel), which is consisted with aforementioned results of PA‐detecting at the PM. Moreover, the signal of Opto‐PASSc in the nucleus was distinctly reduced in response to increasing PLD activities in the cytosol (Figure 1F). This indicates that Opto‐PASSc exhibited a preference for binding to PA rather than entering the nucleus and sequestering them in the nucleus by NLS has limited impact on their ability to bind to PA under conditions where PA levels are extremely elevated in the cytosol.

### 2.2 Light‐Induced Nuclear Export of Opto‐PASSc Enhanced Its Detection Performance in Response to Acute PA Increase

**Figure 2:**
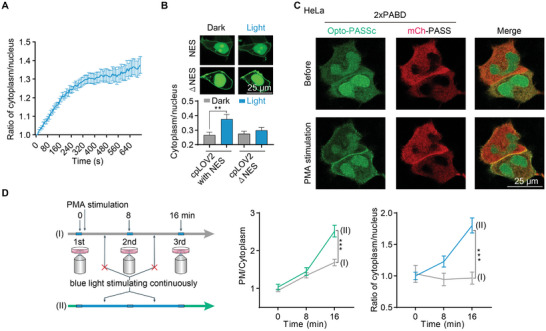
Blue light stimulation induced the nuclear export and enhanced the PA‐detecting ability of Opto‐PASSc biosensor. A) the fluorescence intensity ratio of the cytosol to nucleus was analyzed to indicate the nuclear export of Opto‐PASSc in responsive to blue light stimulation (2% rated power of 488 nm laser, pulse interval was 10 s), the data was normalized to the initial value (*n* ≥ 7 cells). B) The nuclear export of Opto‐PASSc with or without NES (ΔNES) in the Jα helix of the cpLOV2 in responsive to blue light stimulation in 293T cells (*n* ≥ 23 cells). C) Opto‐PASSc and PASS (2×PABD) in detecting PA changes before and after indirect PLD activator PMA (5 × 10^−6^ m) treatment for 16 min. D) The impact of blue light stimulation on the PA‐detecting performance of Opto‐PASSc in response to PMA (5 × 10^−6^ m)‐induced PA production, the condition (I): only three images were captured at indicated time and the interval was dark treatment; the condition (II): blue light stimulation at the capture interval, PMA was added after finishing capture of the first image, the data were normalized to the mean of initial value (*n* ≥ 20 cells). Data were presented as means ± SEM. Two‐tailed Student's *t*‐test is used for (B,D). The *p* values less than 0.05 were considered as significant, *p < 0.05, **p < 0.01, ***p < 0.001.

We first investigated whether blue light‐induced translocation of Opto‐PASSc into the cytosol. The N‐terminal Jα helix fused with the NES was expected to unwind in response to blue light stimulation, exposing the NES and mediating the nuclear export of biosensors. Consequently, more probes were transported into the cytosol for binding to PA. By analyzing the fluorescence intensity ratio of the cytoplasm to the nucleus, we observed an increase in this ratio in response to consecutive blue light pulse stimulation (**Figure** **2**A). Compared to the construct lacking fusion of NES into the Jα helix of cpLOV2, the signals in the nucleus decreased after blue light illumination (Figure 2B), which also suggests the nuclear export of the biosensors.

To evaluate whether the light‐induced export of Opto‐PASSc could enhance its performance in reflecting PA production, we employed an indirect activator, phorbol 12‐myristate 13‐acetate (PMA) (a PKC activator), to instantly increase PA levels. We observed PA levels at the PM was elevated after incubation of PMA (Figure 2C). Considering the short stimulation time of blue light during the acquisition of GFP channel signals, which is insufficient to induce a significant export of the probe from the nucleus, we compared the effects of two treatments: the dark condition and consecutive blue light stimulation at capture intervals, on the performance of Opto‐PASSc biosensor in cells responding to PMA treatment. Our findings revealed that consecutive blue light stimulation at capture intervals reduced the levels of GFP or mCh tagged Opto‐PASSc in the nucleus compared to the dark condition (Figure 2D and Figure S2G, Supporting Information). Moreover, the fluorescence intensity ratio (PM to the cytoplasm) was higher under this condition (Figure 2D and Figure S2G, Supporting Information), suggesting that light illumination enhanced the detection performance of Opto‐PASSc in monitoring instantaneous PA production at the PM.

Collectively, regardless of their expression levels, Opto‐PASSc consistently demonstrates a low background signal by pre‐sequestering unbound probes in the cell nucleus, which provides an accurate reflection of PA levels in the cytosol under basal conditions, a capability not present in the traditional PA biosensor PASS. Its performance was enhanced in detecting PA changes at the PM and mitochondria. The nucleocytoplasmic transport of Opto‐PASSc can be controlled by light according to experimental requirements, indicating greater flexibility in imaging PA in living cells under various experimental conditions. Additionally, this improvement does not compromise the PA‐detecting ability of Opto‐PASSc, as it still preferentially binds to PA rather than extensively entering the nucleus in response to cytoplasmic PA increases.

### 2.3 Development of Optically Controlled Phase Separation to Amplify the Signal of Biosensors

**Figure 3:**
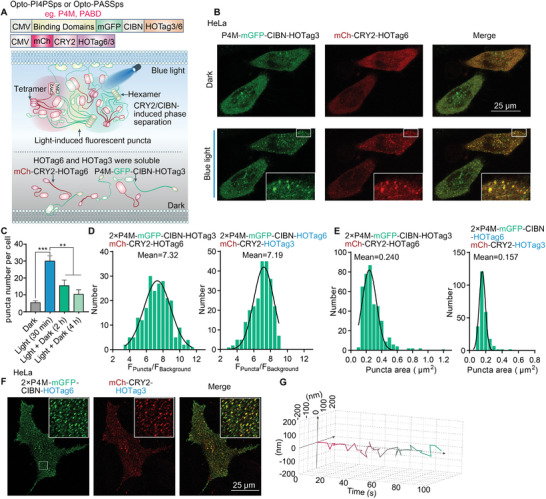
Amplification of biosensors signal based on the optically controlled phase separation. A) A schematic diagram illustrating optically controlled phase separation for optimizing membrane phospholipid biosensors. B) The light‐induced binding of CRY2/CIBN triggered the phase separation via HOTag3/HOTag6 in HeLa cells (blue light stimulation: 2% rated power of 488 nm laser for 10 min and the pulse interval was 5 s). C) The phase separation puncta slowly disaggregated under dark condition (*n* ≥ 40 cells). D) The histogram reflected the brightness distribution of phase separation spots by measuring the fluorescence intensity ratio of puncta to the background in HeLa cells with coexpression of indicated plasmids and E) their the planar dimensions of phase separation puncta (n ≥ 200 puncta). F) The representative images of phase separation puncta depicted the distribution of PI4P on the plasma membrane after light stimulation in HeLa cells and G) the trajectory plot of a representative phase separation punctum. Data were presented as means ± SEM. Two‐tailed Student's *t*‐test is used for (C). The *p* values less than 0.05 were considered as significant, *p < 0.05, **p < 0.01, ***p < 0.001.

Biosensors with high resolution have the potential to significantly advance our understanding of the specific spatiotemporal roles of membrane lipids in live cells. In addition to utilizing super‐resolution microscopy equipment, amplifying the fluorescence signal of biosensors offers an alternative method to visualize the dynamics of probes inside cells. Therefore, we exploited the interaction‐sensitive phase separation of the HOTag3/HOTag6 system to generate punctum‐like structures, thereby amplifying the fluorescence signal of biosensors in cells.^[^ ^19^ ^]^ In this system, HOTag3 can assemble into a hexamer and HOTag6 forms a tetramer. The phase separation would be triggered by the interaction of their fused fragments. By virtue of the light‐responsive binding of the optogenetic tool CRY2/CIBN, the phase separation process of HOTag3/HOTag6 system became controllable via light stimulation (**Figure** **3**A). Then we added a phospholipid‐binding motif, such as PABD and PI4P binding of SidM (P4M) domain, to the CIBN fragment for detecting corresponding phospholipid. In this way, the improved biosensor system generates fluorescent puncta in response to light, and meanwhile the punctum‐like probe can bind to intracellular PA or PI4P, enabling us to visually observe the spatial distribution and precise positional information of PI4P or PA more intuitively. Consequently, this approach improves detection performance compared to the sole expression of the fluorescein‐fused phospholipid‐binding domain in cells (Figure 3A). These improved biosensors were called optogenetic PI4P sensor or PA super sensor based on phase separation (Opto‐PI4PSps or Opto‐PASSps) (Figure 3A).

The results were consistent with our expectations. We observed a 10‐minute light pulse stimulation induced both green and red puncta in cells (Figure 3B), even after we exchanged the HOTag3 and HOTag6 to the corresponding fused proteins (Figure S3A, Supporting Information). To further assess the reversibility of light‐induced phase separation, we incubated the stimulated cells in the absence of light for several hours. Our observations revealed that a portion of the phase separation puncta diffused without the presence of light stimulation (Figure S3B, Supporting Information and Figure 3C). Given that the disassociation time of CRY2 and CIBN is approximately 10–25 min,^[^ ^30^ ^]^ we speculated that the diffusion kinetics of phase separation puncta are primarily determined by the disaggregation of HOTag3 and HOTag6. In addition, we also excluded these puncta were self‐oligomerization of CRY2 (Figure S3C, Supporting Information). Our aforementioned result indicated addition of an extra copy of the P4M domain extremely increased its affinity (Figure S3D, Supporting Information). Consequently, we fused two copies of the P4M domain with CIBN‐HOTag3 or CIBN‐HOTag6. The fluorescence intensity of these puncta was approximately 7.32‐fold higher than the background signal in the GFP channel (Figure 3D). Their planar size was approximately 0.240 µm^2^ (Figure 3E). Intriguingly, upon exchanging the fusion of HOTag3 and HOTag6 with their respective optogenetic counterparts (as indicated in the text), there was a modest reduction in both the brightness and size of these puncta (Figure 3D,E). Additionally, our analysis indicated that the majority of these spots at the PM barely moved^[^ ^14^ ^]^ (Figure 3F,G).

### 2.4 Opto‐PI4PSps and Opto‐PASSps Biosensors Allowed for the Visualization of Intracellular Phospholipids

**Figure 4:**
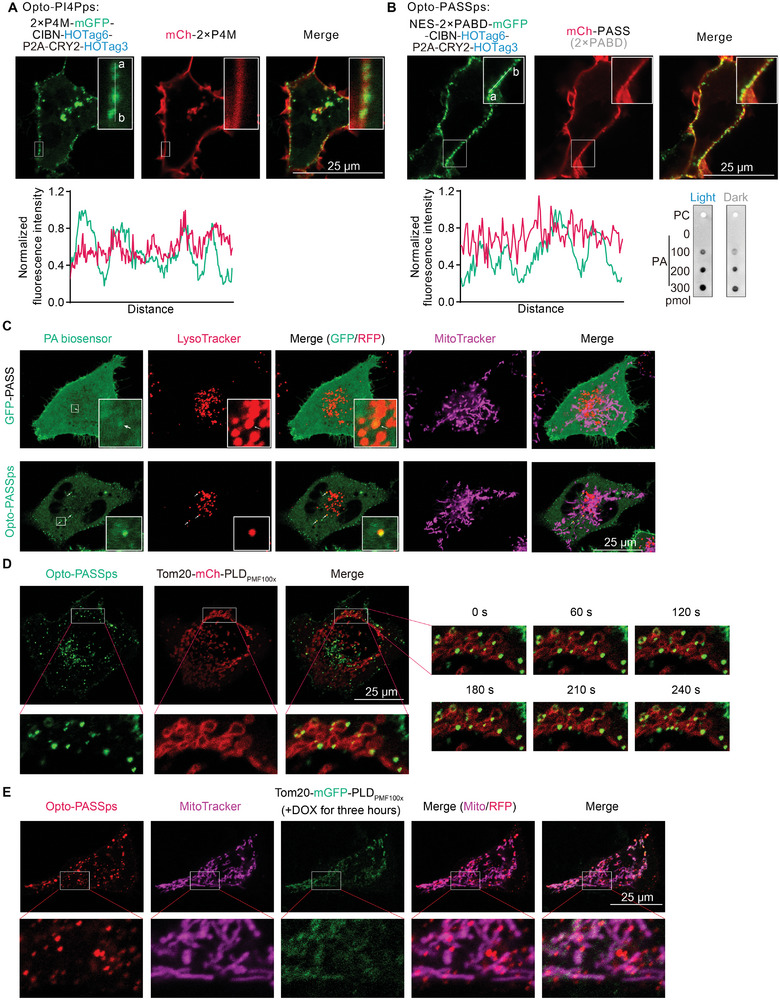
The performance of Opto‐PI4PSps and Opto‐PASSps in detecting intracellular phospholipids. A) A comparison of detecting performance of Opto‐PI4PSps and Opto‐PASSps (B) with the corresponding nonoptimized biosensor after 10 min blue light illumination, respectively; the fluorescence intensity of the white line (ab) was analyzed to assess the amplified signals. The results of PLO assay of Opto‐PASSps were shown in the bottom right panel. Using Opto‐PASSps to detect basal levels of PA on C) the mitochondria and lysosomes and visualize intracellular PA dynamics after overexpression of highly‐active PLD_PMF100×_ on D) the mitochondria in HeLa cells. E) detecting nascent PA production mediated by Dox‐induced PLD_PMF100×_ expression with Opto‐PASSps.

Both Opto‐PI4PSps and Opto‐PASSps biosensors colocalized with the non‐optimized traditional biosensor at the PM (**Figure** **4**A,B). The punctate Opto‐PASSps with mutations in the PABD motif did not colocalize with the control PASS biosensor (Figure S3E, Supporting Information) nor with PLD2 (Figure S3F, Supporting Information). Collectively, these results indicate that phase separation‐mediated signal amplification does not hinder their affinity to the substrates. We also performed a protein lipid overlay (PLO) assay to investigate the impact of phase separation on lipid‐binding ability. The results showed that the punctate PA biosensors induced by light stimulation exhibited enhanced PA‐binding ability compared to the nonstimulated group (Figure 4B, bottom right panel), indicating phase separation increased the affinity of the PA biosensor. We speculated that phase separation incorporates a dozen PABD motifs into a single punctate biosensor. Since the number of PABD copies is positively related to the binding ability of PA biosensors, this incorporation thus enhances their affinity. However, it also indicated an inherent limitation: the exact number of phospholipid molecules bound to a single biosensor was difficult to determine for Opto‐PASSps. It might be regulated by selecting appropriate HOTags to fuse with phospholipid‐binding motifs. For instance, as HOTag6 forms a tetramer, the resulting cluster may have fewer probe heads available for phospholipid binding compared to the hexameric HOTag3.

To investigate whether the punctate distribution of these two biosensors accurately reflects the distribution of PA and PI4P in cells, we used the corresponding inhibitors to pharmacologically deplete PA and PI4P levels. Both biosensors revealed fewer punctate localizations at the PM after inhibitor treatments (Figure S3G,H, Supporting Information). We visually found a time‐dependent reduction in PI4P levels at the PM following treatment with the PI4P inhibitor phenylarsine oxide (PAO) (Figure S3H, Supporting Information). Moreover, with the aid of the optimized probe, we clearly observed that certain punctate probes were co‐localized with lysosomes and moved synchronously with them (Figure S4B and Video S1, Supporting Information). Furthermore, we were able to partially determine the specific location of PI4P on the surface area of the lysosome with Opto‐PI4PSps compared to traditional biosensor (Figure S4A,B, Supporting Information). It has been shown that PI4P would be transferred from lysosomes to mitochondria at their interacting site.^[^ ^31^ ^]^ Through the Opto‐PI4PSps biosensor, we could readily observe that some intracellular PI4P was localized at the interaction site between mitochondria and lysosomes (Figure S4B and Video S1, Supporting Information), suggesting its potential application for tracking phospholipids dynamics at organelle interaction sites. These advantages were not afforded by traditional PI4P biosensors (Figure S4A, Supporting Information).

Due to the relatively low levels of PA under basal conditions and the interference from unbound biosensors, the nonoptimized PASS sensor encountered challenges in detecting small amounts of PA on specific organelles such as lysosomes through colocalization analysis (Figure 4C). However, this situation could be improved by using Opto‐PASSps for detection. PA could be visually observed to co‐localize with lysosomes and exhibit synchronous movement along with them through our improved biosensor (Figure 4C, bottom panel and Video S2, Supporting Information). This observation was consistent with findings using fluorescent dye to label PA, such as IMPACT.^[^ ^25^ ^]^ Furthermore, it has been shown that the proportion of PA in total phospholipids is less than 1% on the mitochondrial membrane.^[^ ^32^ ^]^ In our results, colocalizations of punctum‐like PA biosensors with mitochondria were scarcely observed (Figure 4C), further suggesting a low basal level of PA on the mitochondrial membrane. Increasing PA has been implicated in mitochondrial dynamics and mitophagy^[^ ^33^, ^34^, ^35^, ^36^ ^]^ and addressing its spatiotemporal regulatory mechanisms required high‐performance biosensor. To elevate the PA level on the mitochondria, we overexpressed mitochondria‐localized PLD_PMF100×_, resulting in substantial puncta located on the outer membrane of mitochondria as detected by both GPF‐fused and mCh‐fused Opto‐PASSps biosensor (Figure 4D and Figure S4C, Supporting Information). We also employed DOX to induce PLD_PMF100×_ expression and observed that our enhanced biosensor could detect the PA on the mitochondria induced by low levels of PLD expression (Figure 4E), indicating its high affinity for PA and superior performance in detecting minor PA changes. Interestingly, PA and cardiolipin have been shown to be involved in the regulation of mitochondrial fusion and fission. In this study, we also observed that mitochondrial morphology was altered after the expression of highly active PLD. Previous studies have reported that the overexpression of mitochondrial PLD promotes mitochondrial fusion, suggesting that increased PA might be related to mitochondrial aggregation.^[^ ^35^, ^37^ ^]^ However, we observed more fragmented mitochondria following the increase in PA levels, which is not consistent with those findings. It remains unclear whether this phenomenon is an artificial effect caused by the expression of hyperactive PLD variants or if other unknown regulatory mechanisms are involved.

**Figure 5:**
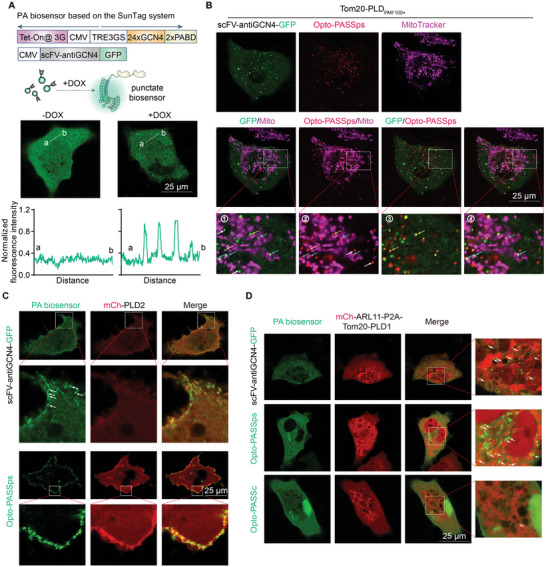
Improving the PA biosensor based on the SunTag system and its comparison with Opto‐PASSps. A) using the SunTag system to improve the PA biosensor, the signal of the line (ab) was normalized to the minimum fluorescence intensity in the line (ab); B) the PA‐detecting performance of PASSst and Opto‐PASSps in PLD‐mediated PA production on the mitochondria, cyan arrow: colocalized PASSst and Opto‐PASSps on the mitochondria, yellow arrow: colocalized PASSst and Opto‐PASSps not on the mitochondria, white arrow in ①: noncolocalized green PASSst biosensor, white arrow in ②: noncolocalized red Opto‐PASSps biosensor. C) PASSst and Opto‐PASSps were compared for their ability to detect PA at the PM in PLD2‐overexpressed HeLa cells. D) PASSst, Opto‐PASSps, and Opto‐PASSc were used to detect PA increases under conditions of ARL11‐induced PLD1 activation.

Currently, the SunTag system is also a powerful tool for amplifying signals by fusion of multicopy peptides into the target molecule. These short peptides could be recognized by the single‐chain variable fragments (scFV) antibody or nanoantibody. The resulting clusters resemble punctum‐like shapes that amplify single‐molecular signals.^[^ ^14^ ^]^ In this system, it is crucial to control the number of fluorescent clusters to prevent a significant amount of dissociated probes from affecting the visualization of puncta. Therefore, we commonly used tetracycline to induce the expression of the target molecule. To investigate the potential of the SunTag system in enhancing biosensor performance, we fused two copies of PABDs with the tandem GCN4 peptides (24 × GCN4) (here referred to as PASS based on the SunTag system (PASSst)) and coexpressed them with anti‐GCN4 scFV antibody probes in HeLa cells. After the addition of DOX, punctum‐like structures were clearly observed (**Figure** **5**A). These puncta exhibited a size similar to the aforementioned optogenetic biosensors but displayed a lower brightness ratio (the ratio of spot brightness to the background) (Figure 5A). We coexpressed PASSst and Opto‐PASSps in cells overexpressing Tom20‐PLD_PMF100×_. The red Opto‐PASSps spots were partially colocalized with green punctate PASSst, and some of these puncta were located on the mitochondria (Figure 5B ④ cyan arrows), which suggests PLD‐induced PA production and also indicates the spatial location of PA on the mitochondria. However, a small set of the colocalized PA biosensors (yellow spots) were not on the mitochondria and moved faster than mitochondrial spots in cells (Figure 5B ③ yellow arrows). As mentioned earlier, there were undeniable levels of PA on the lysosomes, leading us to suspect that these spots might label the PA present on the lysosomes.

In addition, we assessed the performance of PASSst and Opto‐PASSps in detecting the activities of wild‐type mammalian PLDs. In PLD2‐overexpressing HeLa cells, both probes were mainly localized at the plasma membrane and colocalized with PLD2 (Figure 5C). A recent study demonstrated that ADP‐ribosylation factor‐like protein 11 (ARL11) is a novel potent activator of PLD1 through its interaction with the PLD1 loop region. While this activation has been well‐demonstrated in vitro, its cellular activities have not been revealed. To investigate this, we constructed a mCh‐ARL11‐P2A‐Tom20‐PLD1 plasmid to observe the colocalization of ARL11 and PLD1 and detect the PA levels on the mitochondria resulting from ARL11‐induced PLD1 activation. The results showed that mCh‐labeled ARL11 was partially localized on the mitochondria compared to an inactivated mutant ARL11 (Q67L) (Figure S4E, Supporting Information), further indicating its interaction with PLD1. Both PASSst and Opto‐PASSps showed an increase in PA on the mitochondria, whereas Opto‐PASSc did not perform as well as the other two (Figure 5D), suggesting the advantage of punctate biosensors in detecting minor PA changes.

Taken together, the strategy of optically‐controlled phase separation generated a similar amplification of the fluorescence signal of the biosensors in comparison to the SunTag system, but distinguished themselves by their light‐controllable puncta formation rather than drug‐induced expression. The brightness of punctate biosensors also provided protection against photobleaching during prolonged exposure, unlike traditional biosensors. Moreover, phase separation‐mediated punctate biosensors exhibited increased substrate affinity. These punctum‐like structures allowed for the visualization of intracellular phospholipid distribution and monitoring their minor dynamic changes, which provide a convenient approach to investigate the spatiotemporal regulatory roles of phospholipids in live cells.

## 3 Conclusion

In summary, our study represents a significant advancement in utilizing optogenetic strategies to enhance the functionality of phospholipid biosensors. Compared to traditional approaches in biosensor development, these optogenetics‐based enhancements effectively reduce background signals, providing clearer reflections of phospholipid changes in cells. The application of optically‐controlled phase separation induces the formation of fluorescent spots, amplifying signals and facilitating phospholipids visualization in cells. The improved biosensors presented in our study enriched the toolbox for phospholipid imaging and they hold great potential for enhancing our understanding of the spatiotemporal dynamics and regulatory roles of membrane lipids in living cells. The methodological improvements outlined in this study offer valuable insights for the development of other high‐performance biosensors.

## 4 Experimental Section

### Construction of Optogenetics‐Based Biosensors

For cpLOV2‐based sensor, cpLOV2 sequence of which the Jα helix contained a NES sequence was first synthesized. To replace different NES in the Jα helix of cpLOV2, two BsmBI sites into both ends of NES and fused with mCherry‐NLS. These fragments were subcloned into pLenti‐MCS‐GFP‐SV40‐Puro empty vector that linearized by XbaI and SpeI restriction enzymes (New England Biolabs, Inc., USA) via Gibson Assembly kit (TransGen Biotech., China). This framework (pLenti‐cpLOV2‐mCherry‐NLS) was further modified by the replacement of mCherry‐NLS with NLS‐mGFP‐NES‐PABP, namely Opto‐PASSc in this study. The sequence of mGFP‐NES‐PABP was amplified from GFP‐PASS, a gift from Prof. Guangwei Du. Red Opto‐PASSc probe was also constructed by the replacement of mGFP with mCherry sequence. PABP 4E mutations were introduced into the PABP sequence by primers and its PCR fragment, together with cpLOV2‐NLS‐mCherry‐NES fragment, was inserted into corresponding linearized pLenti‐MCS‐GFP‐SV40‐Puro.

The PI4P sensor based on optically controlled phase separation was created by fusion of P4M domain from mCherry‐P4M‐SidM (#51471, Addgene, USA) to mGFP‐CIBN. The mGFP (Emerald) was amplified from Tom20‐Emerald (#54282, Addgene, USA) and the CIBN was amplified from CIBN‐CAAX (#79574, Addgene, USA). The CRY2 was amplified from the mCherry‐CRY2‐PR variant^[^ ^38^ ^]^ and the linker between mCherry and CRY2 was generated by the primer (5′‐AGATCTCGAAGCGCGGCCGCGGGAGCAGGAGGAGCAGCTCGAGCTATGAAG‐3′). HOTag3 and HOTag6 was synthesized (Beijing Tsingke Biotech Co., Ltd. China) with corresponding homologous sequence in both ends and a sequence encoding a specific linker. They were subcloned into linearized vector with (P4M)*_n_*‐mGFP‐CIBN and mCh‐CRY2, respectively. The PLD_PMF100×_ was synthesized (Beijing Tsingke Biotech Co., Ltd. China) according to Baskin's publication.^[^ ^29^ ^]^ The PASSst sensor was created by fusion of two copies of PABD with the GCN4 that was amplified from pcDNA4TO‐24xGCN4_v4‐kif18b (#74934, Addgene, USA). The GCN4 probe was pHR‐scFv‐GCN4‐sfGFP‐GB1‐dWPRE (#60907, Addgene, USA). The above plasmids were assembled with the corresponding purified PCR fragments via Gibson Assembly kit. The sequence and source of other constructions used in this study are listed in Supporting Information and all plasmids used in this study were sequenced for validation.

### Cell Culture and Transfection

Cancer cell lines HeLa and 293T used in this research were purchased from ATCC. 293T cell were routinely grown in Dulbecco's modified essential media (DMEM, Hyclone), while HeLa cells were routinely grown in RPMI 1640. The complete medium was supplemented with 10% fetal bovine serum (PAN, PAN‐Biotech, Germany) and 100 U mL^−1^ penicillin/streptomycin (Beyotime Biotechnology, China). The cells were cultured at 37 °C in a humidified atmosphere with 5% CO_2_. For transient over‐expression assay, the cells were plated on confocal dishes. About 1–2 µg indicated plasmid was used according to the instruction of lipofectamine 3000 (Thermo Fisher Scientific, USA).

### Living Cell Imaging

Cells were plated on the confocal dish and then were cotransfected with indicated plasmid. After 24 h transfection, refreshed with the phenol‐free medium. The confocal images were obtained using a AX confocal laser scanning microscope (Nikon, Japan) equipped with a 60×/1.49 numerical aperture (NA) oil immersion objective lens. For light‐induced PA probe export, the blue light pulse was 10–12 s and the 488 laser power was 1 mW. For the time‐scale detection of PA at plasma membrane, such as PMA‐induced PA production, the first image was captured under nonstimulation condition and subsequently 10 µL PBS buffer containing 500 × 10^−6^ m PMA (P8139, Sigma‐Aldrich, USA) was added into the dish carefully. The capture interval was 8 minutes. For time‐scale detection of PI4P at plasma membrane, the images were captured by a C2 confocal laser scanning microscope with a TIRF module (Nikon, Japan). The first image was captured and subsequently PAO (P3075, Sigma‐Aldrich, USA) with the indicated final concentration was added into the dish carefully. The capture interval was 10 min.

### The Protein Lipid Overlay (PLO) Assay

The PLO assay was performed as described in a previous study.^[^ ^39^ ^]^ Briefly, Opto‐PASSps was transfected into 293T cells. After a 1 h blue light treatment, cells from both the control group (dark treatment) and the light‐stimulated group were lysed in a buffer containing 140 × 10^−3^ m NaCl, 10 × 10^−3^ m HEPES, 1 × 10^−3^ m EDTA, 1% NP‐40, and 1 × 10^−3^ m PMSF. PA (P3075, MERCK, USA) was dissolved in methanol at a concentration of 40 mM and diluted in a 2:1:0.8 solution of methanol:chloroform:water to three different concentrations ranging between 100 × 10^−6^ and 300 × 10^−6^ m. A 1 µL aliquot of each dilution was spotted onto a NC membrane (HATF00010, 0.45 µm pore size, Millipore, USA). The membrane was blocked in TBST containing 3% BSA, and the Opto‐PASSps lysate (0.5 mg mL^−1^, diluted with blocking buffer) was used to incubate the membrane for 3 h at room temperature. Anti‐GFP monoclonal antibody (M20004, Abmart, China) was used to recognize the Opto‐PASSps biosensor. The membranes from both the dark and light groups were detected simultaneously using ECL according to the manufacturer's instructions.

### Statistical Analysis

The fluorescence intensity of the images was analyzed by FiJi Image J software (Version 1.53t). Two experimental groups were analyzed by unpaired Student's *t*‐test (two‐tailed). Multigroup comparisons were performed by the one‐way ANOVA with Tukey's post hoc test. The *p* values less than 0.05 were considered as significant, *p < 0.05, **p < 0.01, ***p < 0.001. Quantitative data are presented as mean ± SEM. Details of the *n* number can be found in the appropriate figure legend. GraphPad Prism was used for data analysis and plot.

## Conflict of Interest

The authors declare no conflict of interest.

## Author Contributions

Y.Y. and X.L. contributed equally to this work. Y.Y., X.L., and L.H. conceived the idea of optogenetic strategies for improvement of biosensors and discussed the optimization of opto‐biosensors. H.L., L.H., and Y.X. supervised the research. Y.Y. and X.L. designed and conducted most of the experiments. J.L., W.S., Y.C., S.C., T.Z., and S.K. prepared the samples. Y.Y., X.L., J.L., W.S., and Y.C. analyzed the experimental data and plotted figures. Y.Y., H.L., L.H., and Y.X. wrote and revised the paper with input from all authors.

## Supporting information

Supporting Information

Supplemental Video 1

Supplemental Video 2

Supplemental Video 3

## Data Availability

Research data are not shared.
