# Supplementary material for: Optogenetic Strategies for Optimizing the Performance of Phospholipids Biosensors
Source: Adv Sci (Weinh). 2024 Jul 29;11(36):2403026. doi: 10.1002/advs.202403026 (PMC11422808; doi:10.1002/advs.202403026)
Supplement: Supplementary file 1 — Supporting Information [file ADVS-11-2403026-s002.docx]

**Supplementary Material for:**

**Optogenetic Strategies for Optimizing the Performance of Phospholipids Biosensors**

Yuanfa Yao^1, #,^, Xiayan Lou^1, #^, Luhong Jin^2^, Weiyun Sun^1^, Jingfang Liu^3^, Yunyue Chen^3^, Sunying Cheng^1^, Tengjiao Zhao^1^, Shuwei Ke^1^, Luhao Zhang^2^, Yingke Xu^3, 4 *^, Lian He^5, *^, Hanbing Li^1, *^

*1 Institute of Pharmacology, College of Pharmaceutical Science, Zhejiang University of Technology, Hangzhou, 310014, China*

*2 School of Information Science and Technology, Hangzhou Normal University, Hangzhou, China*

*3 Department of Biomedical Engineering, Key Laboratory of Biomedical Engineering of Ministry of Education, State Key Laboratory of Extreme Photonics and Instrumentation, Zhejiang Provincial Key Laboratory of Cardio-Cerebral Vascular Detection Technology and Medicinal Effectiveness Appraisal, Zhejiang University, Hangzhou, 310027, China*

*4 Department of Endocrinology, Children’s Hospital of Zhejiang University School of Medicine, National Clinical Research Center for Children’s Health, Hangzhou, Zhejiang 310051, China*

*5 Department of Pharmacology, School of Medicine, Southern University of Science and Technology, Shenzhen, 518055, China.*

# These authors contributed equally: Yuanfa Yao, Xiayan Lou

*Correspondence should be addressed to **Drs. Hanbing Li**, **Drs.** **Lian He**, **Prof.** **Yinek Xu**,

**Address for Dr. Hanbing Li (lead):** Institute of Pharmacology, College of Pharmaceutical Science, 18 Chaowang Road, Zhejiang University of Technology, Hangzhou, 310014, China

Email: hanbing.li@163.com; Tel & Fax: +86-13185064285

**Address for Dr. Lian He:** Department of Pharmacology, School of Medicine, 1088 Xueyuan Avenue, Southern University of Science and Technology, Shenzhen, 518055, China.

Email: hel3@sustech.edu.cn; Tel & Fax:+86-0755-880100

**Address for Prof. Yingke Xu:** Department of Biomedical Engineering, 38 Zheda Road, Yuquan Campus, Zhejiang University, Hangzhou, 310027, China

Email: yingkexu@zju.edu.cn ; Tel & Fax: +86-0571-87951091

1. **Supplementary figures (1-4)**

**
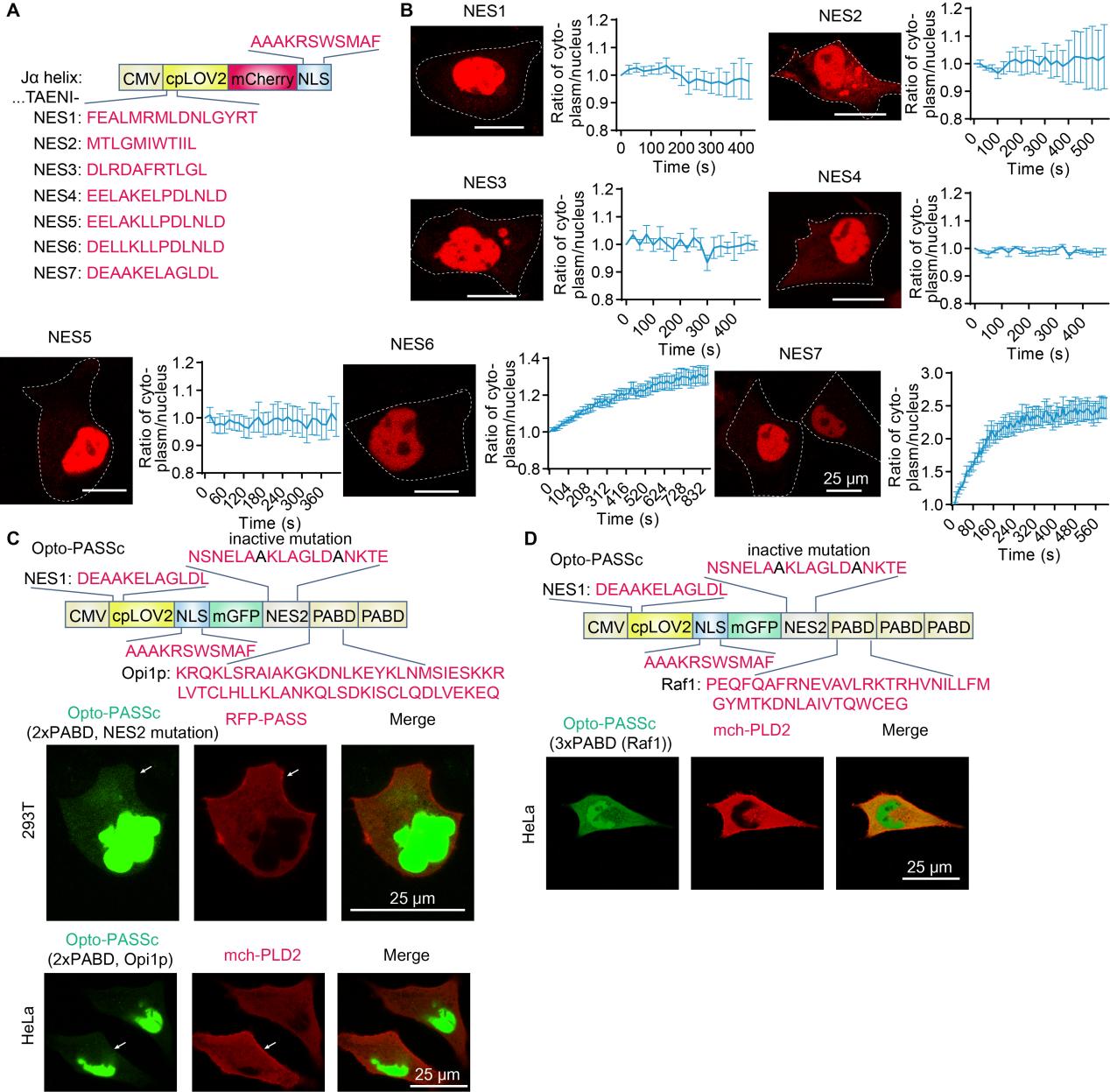
**

**Supplementary figure S1** **Screening of NES for regulating the nuclear export of cpLOV2 and evaluation of the PA binding motif among various PA binding proteins. A**, the design of cpLOV-based nucleocytoplasmic transportation and **B**, representative images of cpLOV-mCh-NLS distribution in cells under non-stimulation condition and quantitative analysis of the light-induced nuclear export of cpLOV-mCh-NLS with fusion of different NES sequences. **C**, the schematic diagram of the cpLOV-based PA biosensor (Opto-PASSc) with two PABD motifs from yeast Opi1p and representative images of co-expression of Opto-PASSc with RFP-PASS or mCh-PLD2 in HeLa cells and in 293T cells. **D**, the schematic diagram of the cpLOV-based PA biosensor (Opto-PASSc) with three PABD motifs from Raf1 protein and representative images of its co-expression with mCh-PLD2.

**
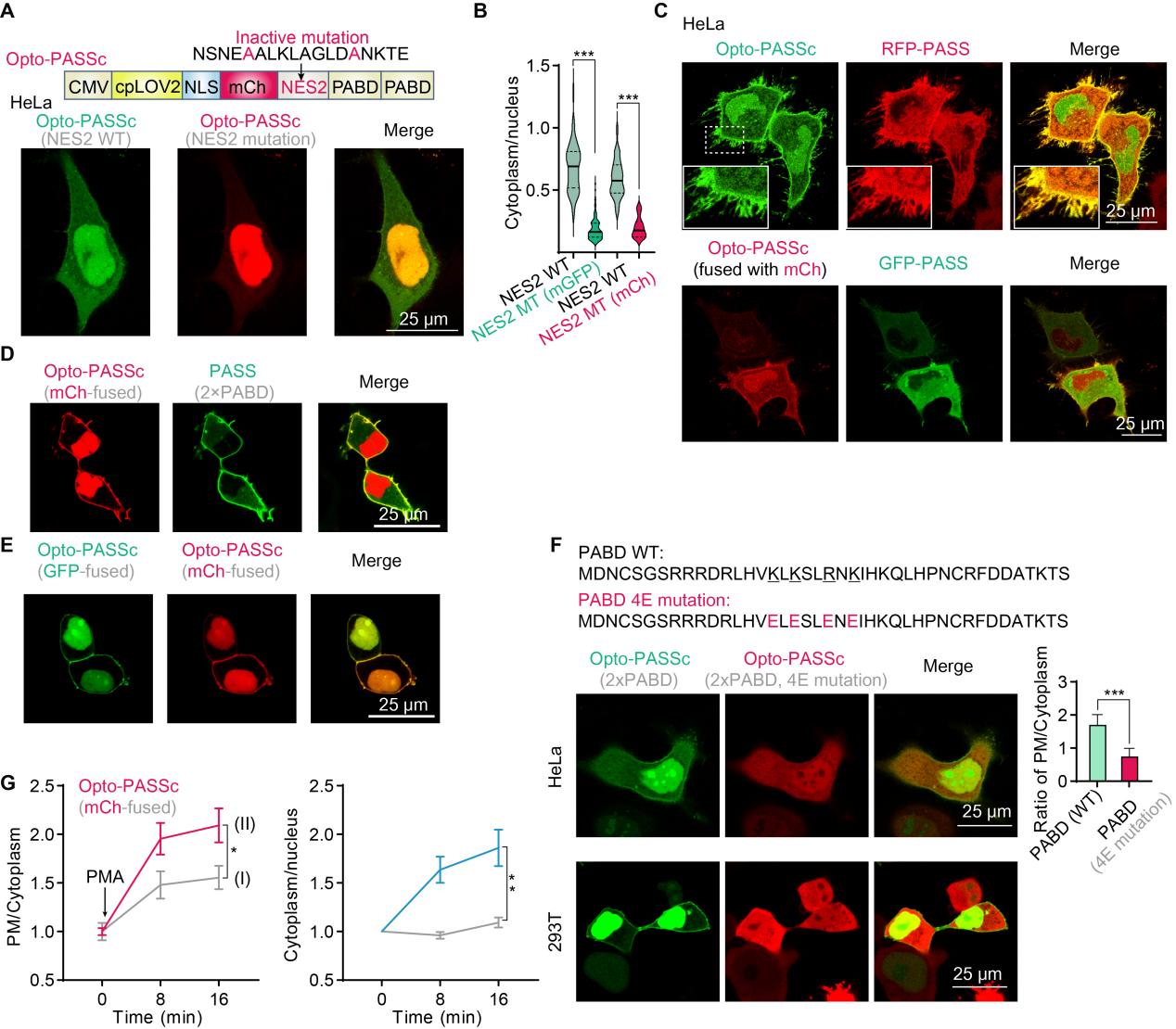
**

**Supplementary figure S2 Optimization of Opto-PASSc in** **background noise and its comparison with the wildly-used PASS. A**, further reducing the background noise from the unbound PASS by inactive mutation of internal NES2, two residues (Leucine and Isoleucine) of the NES2 was mutated into Alanine and colored in red. **B**, assessing the improvement of the background signal after NES2 mutations by measuring the fluorescence intensity ratio of the cytosol to the nucleus , this analysis involved comparing the following groups: wild-type NES2 vs. NES2 with mutations (mGFP-fused) (n ≥ 70 cells) and wild-type NES2 vs. NES2 with mutations (mCherry-fused) (n = 27 cells). **C**, the colocalization of Opto-PASSc (fused with mGFP or mCherry) with the wildly-used PASS biosensor in HeLa cells. **D**, representative images of co-expression of the mCh-fused Opto-PASSc with the GFP-PASS containing the same copy of PABD, and **E**, representative images of co-expression of Opto-PASSc (2×PABD) fused with GFP or mCh. **F**, amino acid sequence of PABD and 4E mutant PABD and representative images of Opto-PASSc with or without 4E mutation in HeLa cells and in 293T cells. The ratio of plasma membrane to cytosol were analyzed in HeLa (n=11 cells). **G**, the impact of blue light stimulation on the PA-detecting performance of mCh-fused Opto-PASSc in response to PMA (5 μM)-induced PA production, the condition (I): only three images were captured at indicated time and the interval was dark treatment; the condition (II): blue light stimulation at the capture interval, PMA was added after finishing capture of the first image, the data was normalized to the initial value (n = 7 cells). The PM/Cytoplasm ratio was normalized to the mean of the initial value, and the data of Cytoplasm/nucleus was normalized to their respective initial values. Data were presented as means ± SEM. Two-tailed Student’s *t* test is used for (F, G). The *p* values less than 0.05 were considered as significant, **p* < 0.05, ***p* < 0.01, ****p* < 0.001.

**
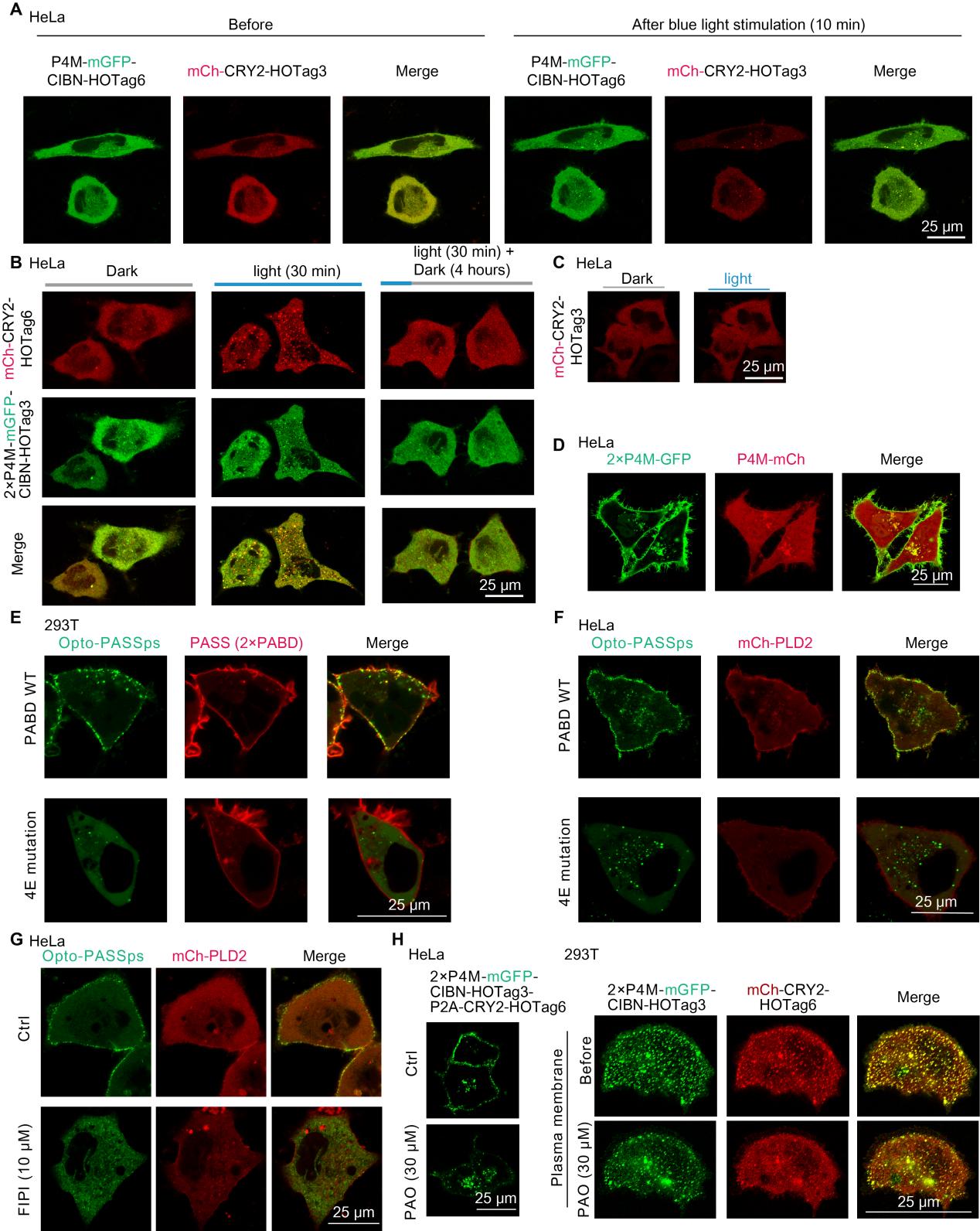
**

**Supplementary figure S3 The optically-controlled phase separation amplified the signal of PI4P biosensor and its application in detecting PI4P changes after chemical inhibitor treatment.** **A**, the light-induced binding of CRY2/CIBN triggered the phase separation via HOTag3/HOTag6 in HeLa cells with co-expression of indicated plasmids. **B**, the disaggregation of the light-induced phase separation after dark treatment. **C**, the phase separation did not induced by light in the cells with single expression of mCh-CRY2-HOTag3. **D**, the representative image of PI4P biosensors with one or two copies of P4M. **E**, co-expression of the wild-type PABD or 4E mutation (unable to binds to PA) with PASS (PABD WT) in 239T cells. **F**, co-expression of the wild-type PABD or 4E mutation with PLD2. **G**, Opto-PASSps was employed to detect PA changes on PM in HeLa cells after a four-hour FIPI (10 μM) treatment. **H**, the enhanced probe Opto-PI4PSps was employed to detect PI4P changes on PM in HeLa cells, and the TIRF imaging was used to monitor PI4P changes on PM in 293T cells in respond to a half-hour treatment with a PI4P inhibitor, PAO (30 μM).

**
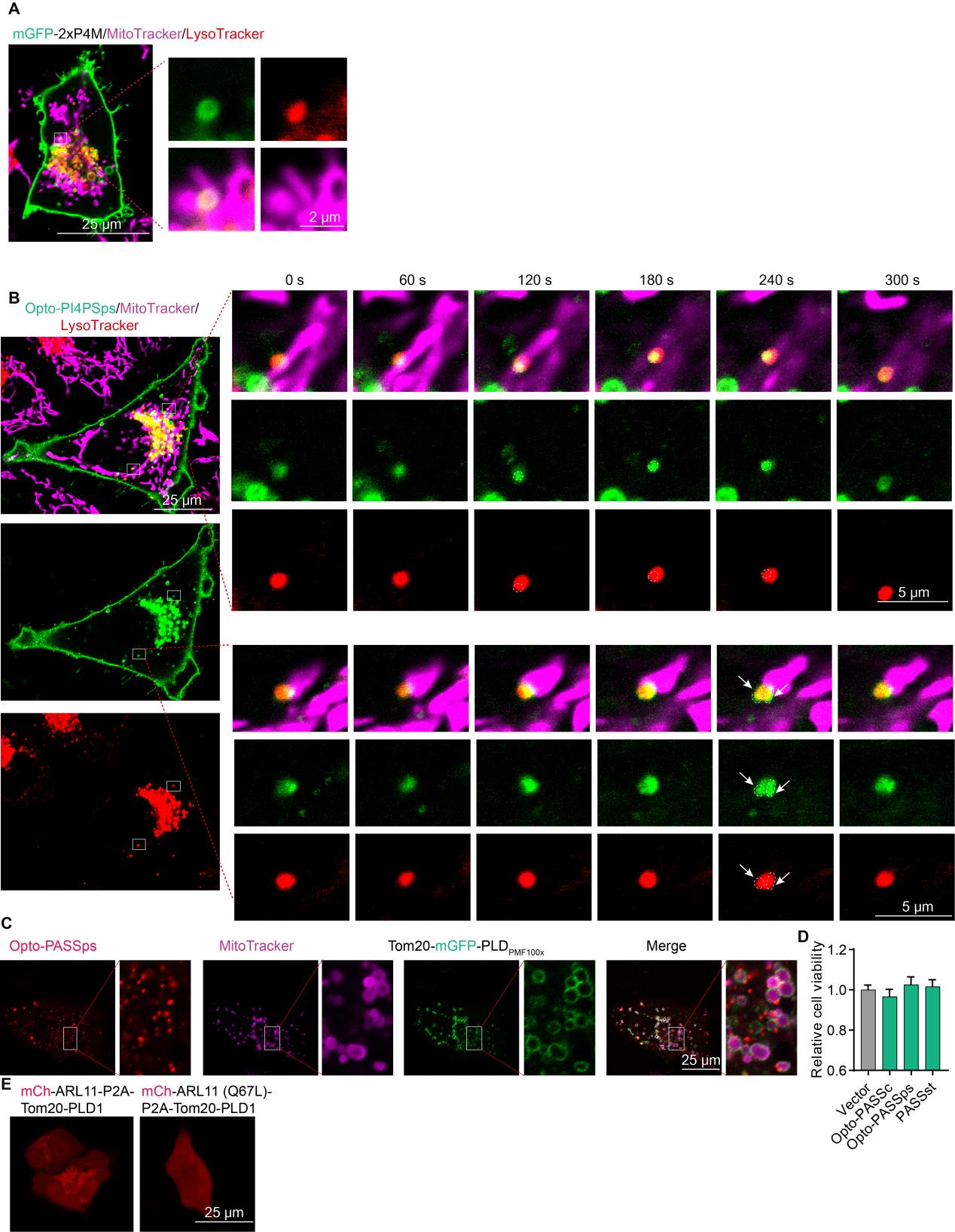
**

**Supplementary figure S4** A comparison of mGFP-2×P4M (**A**) with Opto-PI4PSps (**B**) in visualizing intracellular PI4P. PI4P was localized on some of lysosomes through the non-optimized PI4P biosensor, while Opto-PI4PSps visually and informatively presented PI4P on the lysosomes and the interacting site of lysosome and mitochondria. **C**, Using mCh-fused Opto-PASSps to visualize intracellular PA dynamics after overexpression of highly-active PLD_PMF100×_ on the mitochondria. **D**, the cell viability of 293T cells after overexpression of three optimized PA biosensor (n ≥ 8) . **E**, Co-expression of human PLD1 with ARL11 wild-type or variant Q67L in HeLa cells. Data were presented as means ± SEM. Two-tailed Student’s *t* test is used for (D). The *p* values less than 0.05 were considered as significant, **p* < 0.05, ***p* < 0.01, ****p* < 0.001.

**2. sequence of plasmids used in this study.**

| Plasmid | Sequence |
| --- | --- |
| pLenti-CMV-cpLOV2(NES1 in the Jα helix)  -NLS-mGFP(or mCh)  -NES2(MT or WT)-  2×PABD(or 4×PABD) | cpLOV2: yellow linker: red NLS: blue   mGFP: green  NES1: gray PABP: deep yellow NES2: gray  MTEHVRDAAEREGVMLIKKTAENIDEAAKELAGLDLGGGSGGSGGGLATTLERIEKNFVITDPRLPDNPIIFASDSFLQLTEYSREEILGRNCRFLQGPETDRATVRKIRDAIDNQTEVTVQLINYTKSGKKFWNLFHLQPMRDQKGDVQYFIGVQLDGGSGSGSAAAKRSWSMAFGSGSGSMVSKGEELFTGVVPILVELDGDVNGHKFSVSGEGEGDATYGKLTLKFICTTGKLPVPWPTLVTTLTYGVQCFSRYPDHMKQHDFFKSAMPEGYVQERTIFFKDDGNYKTRAEVKFEGDTLVNRIELKGIDFKEDGNILGHKLEYNYNSHNVYIMADKQKNGIKVNFKIRHNIEDGSVQLADHYQQNTPIGDGPVLLPDNHYLSTQSKLSKDPNEKRDHMVLLEFVTAAGITLGMDELYKSGLRSRANSNE**A**ALKLAGLD**A**NKTESRMDNCSASRRRDRLHVKLKSLRNKIHKQLHPNCRFDDATKTSGGGSGGGSMDNCSGSRRRDRLHVKLKSLRNKIHKQLHPNCRFDDATKTS  **NES2WT:** NSNELALKLAGLDINKTE  **mCherry:** MVSKGEEDNMAIIKEFMRFKVHMEGSVNGHEFEIEGEGEGRPYEGTQTAKLKVTKGGPLPFAWDILSPQFMYGSKAYVKHPADIPDYLKLSFPEGFKWERVMNFEDGGVVTVTQDSSLQDGEFIYKVKLRGTNFPSDGPVMQKKTMGWEASSERMYPEDGALKGEIKQRLKLKDGGHYDAEVKTTYKAKKPVQLPGAYNVNIKLDITSHNEDYTIVEQYERAEGRHSTGGMDELYK  **4×PABD:**  MDNCSASRRRDRLHVKLKSLRNKIHKQLHPNCRFDDATKTSGGGSGGGSMDNCSGSRRRDRLHVKLKSLRNKIHKQLHPNCRFDDATKTSGSGSGSGSANKTESRMDNCSASRRRDRLHVKLKSLRNKIHKQLHPNCRFDDATKTSGGGSGGGSMDNCSGSRRRDRLHVKLKSLRNKIHKQLHPNCRFDDATKTS |
| pLenti-mGFP(mCh)-NES2(WT)-2×PABD (or 4×PABD) | linker: red NLS: blue mGFP: green NES2: deep gray PABP: deep yellow  MVSKGEELFTGVVPILVELDGDVNGHKFSVSGEGEGDATYGKLTLKFICTTGKLPVPWPTLVTTLTYGVQCFSRYPDHMKQHDFFKSAMPEGYVQERTIFFKDDGNYKTRAEVKFEGDTLVNRIELKGIDFKEDGNILGHKLEYNYNSHNVYIMADKQKNGIKVNFKIRHNIEDGSVQLADHYQQNTPIGDGPVLLPDNHYLSTQSKLSKDPNEKRDHMVLLEFVTAAGITLGMDELYKSGLRSRANSNELALKLAGLDINKTESRMDNCSASRRRDRLHVKLKSLRNKIHKQLHPNCRFDDATKTSGGGSGGGSMDNCSGSRRRDRLHVKLKSLRNKIHKQLHPNCRFDDATKTS  **mCherry:** MVSKGEEDNMAIIKEFMRFKVHMEGSVNGHEFEIEGEGEGRPYEGTQTAKLKVTKGGPLPFAWDILSPQFMYGSKAYVKHPADIPDYLKLSFPEGFKWERVMNFEDGGVVTVTQDSSLQDGEFIYKVKLRGTNFPSDGPVMQKKTMGWEASSERMYPEDGALKGEIKQRLKLKDGGHYDAEVKTTYKAKKPVQLPGAYNVNIKLDITSHNEDYTIVEQYERAEGRHSTGGMDELYK  **4×PABD:**  MDNCSASRRRDRLHVKLKSLRNKIHKQLHPNCRFDDATKTSGGGSGGGSMDNCSGSRRRDRLHVKLKSLRNKIHKQLHPNCRFDDATKTSGSGSGSGSANKTESRMDNCSASRRRDRLHVKLKSLRNKIHKQLHPNCRFDDATKTSGGGSGGGSMDNCSGSRRRDRLHVKLKSLRNKIHKQLHPNCRFDDATKTS |
| pLenti-2×P4M-GFP-  CIBN-HOTag3 | P4M: deep yellow GFP: green linker: red CIBN: black linker-HOTag3: blue  MTASTENFKNVKEKYQQMRGDALKTEILADFKDKLAEATDEQSLKQIVAELKSKDEYRILAKGQGLTTQLLGLKTSSVSSFEKMVEETRESIKSQERQTIKIKGGGSGGGSTASTENFKNVKEKYQQMRGDALKTEILADFKDKLAEATDEQSLKQIVAELKSKDEYRILAKGQGLTTQLLGLKTSSVSSFEKMVEETRESIKSQERQTIKIKGGMVSKGEELFTGVVPILVELDGDVNGHKFSVSGEGEGDATYGKLTLKFICTTGKLPVPWPTLVTTLTYGVQCFARYPDHMKQHDFFKSAMPEGYVQERTIFFKDDGNYKTRAEVKFEGDTLVNRIELKGIDFKEDGNILGHKLEYNYNSHKVYITADKQKNGIKVNFKTRHNIEDGSVQLADHYQQNTPIGDGPVLLPDNHYLSTQSKLSKDPNEKRDHMVLLEFVTAAGITLGMDELYASGSGSMNGAIGGDLLLNFPDMSVLERQRAHLKYLNPTFDSPLAGFFADSSMITGGEMDSYLSTAGLNLPMMYGETTVEGDSRLSISPETTLGTGNFKAAKFDTETKDCNEAAKKMTMNRDDLVEEGEEEKSKITEQNNGSTKSIKKMKHKAKKEENNFSNDSSKVTKELEKTDYIHVGSGSAGGSAGGSAGGSAGGSAGGSAGGSAGGSRGEIAKSLKEIAKSLKEIAWSLKEIAKSLKG |
| pLenti-mCh-CRY2-  HOTag6 | mCh: deep red CRY2: deep blue linker: red linker-HOTag6: blue  MVSKGEEDNMAIIKEFMRFKVHMEGSVNGHEFEIEGEGEGRPYEGTQTAKLKVTKGGPLPFAWDILSPQFMYGSKAYVKHPADIPDYLKLSFPEGFKWERVMNFEDGGVVTVTQDSSLQDGEFIYKVKLRGTNFPSDGPVMQKKTMGWEASSERMYPEDGALKGEIKQRLKLKDGGHYDAEVKTTYKAKKPVQLPGAYNVNIKLDITSHNEDYTIVEQYERAEGRHSTGGMDELYKRSRSAAAGAGGAARAMKMDKKTIVWFRRDLRIEDNPALAAAAHEGSVFPVFIWCPEEEGQFYPGRASRWWMKQSLAHLSQSLKALGSDLTLIKTHNTISAILDCIRVTGATKVVFNHLYDPVSLVRDHTVKEKLVERGISVQSYNGDLLYEPWEIYCEKGKPFTSFNSYWKKCLDMSIESVMLPPPWRLMPITAAAEAIWACSIEELGLENEAEKPSNALLTRAWSPGWSNADKLLNEFIEKQLIDYAKNSKKVVGNSTSLLSPYLHFGEISVRHVFQCARMKQIIWARDKNSEGEESADLFLRGIGLREYSRYICFNFPFTHEQSLLSHLRFFPWDADVDKFKAWRQGRTGYPLVDAGMRELWATGWMHNRIRVIVSSFAVKFLLLPWKWGMKYFWDTLLDADLECDILGWQYISGSIPDGHELDRLDNPALQGAKYDPEGEYIRQWLPELARLPTEWIHHPWDAPLTVLKASGVELGTNYAKPIVDIDTARELLAKAISRTREAQIMIGAAARGAAAGAGGAGRGGGGSGSGSAGGSAGGSAGGSAGGSAGGSAGGSAGGSRTLREIEELLRKIIEDSVRSVAELEDIEKWLKKI |
| pLenti-P4M (or 2×P4  M)-GFP-CIBN-HOTag6 | P4M: deep yellow GFP: green CIBN: black linker-HOTag6: blue  MTASTENFKNVKEKYQQMRGDALKTEILADFKDKLAEATDEQSLKQIVAELKSKDEYRILAKGQGLTTQLLGLKTSSVSSFEKMVEETRESIKSQERQTIKIKGGMVSKGEELFTGVVPILVELDGDVNGHKFSVSGEGEGDATYGKLTLKFICTTGKLPVPWPTLVTTLTYGVQCFARYPDHMKQHDFFKSAMPEGYVQERTIFFKDDGNYKTRAEVKFEGDTLVNRIELKGIDFKEDGNILGHKLEYNYNSHKVYITADKQKNGIKVNFKTRHNIEDGSVQLADHYQQNTPIGDGPVLLPDNHYLSTQSKLSKDPNEKRDHMVLLEFVTAAGITLGMDELYASGSGSMNGAIGGDLLLNFPDMSVLERQRAHLKYLNPTFDSPLAGFFADSSMITGGEMDSYLSTAGLNLPMMYGETTVEGDSRLSISPETTLGTGNFKAAKFDTETKDCNEAAKKMTMNRDDLVEEGEEEKSKITEQNNGSTKSIKKMKHKAKKEENNFSNDSSKVTKELEKTDYIHVGSGSAGGSAGGSAGGSAGGSAGGSAGGSAGGSRTLREIEELLRKIIEDSVRSVAELEDIEKWLKKI  **2×P4M:**  MTASTENFKNVKEKYQQMRGDALKTEILADFKDKLAEATDEQSLKQIVAELKSKDEYRILAKGQGLTTQLLGLKTSSVSSFEKMVEETRESIKSQERQTIKIKGGGSGGGSTASTENFKNVKEKYQQMRGDALKTEILADFKDKLAEATDEQSLKQIVAELKSKDEYRILAKGQGLTTQLLGLKTSSVSSFEKMVEETRESIKSQERQTIKIK |
| pLenti-mCh-CRY2-  HOTag3 | mCherry:deep red CRY2:deep blue linker:red linker-HOTag3: blue  MVSKGEEDNMAIIKEFMRFKVHMEGSVNGHEFEIEGEGEGRPYEGTQTAKLKVTKGGPLPFAWDILSPQFMYGSKAYVKHPADIPDYLKLSFPEGFKWERVMNFEDGGVVTVTQDSSLQDGEFIYKVKLRGTNFPSDGPVMQKKTMGWEASSERMYPEDGALKGEIKQRLKLKDGGHYDAEVKTTYKAKKPVQLPGAYNVNIKLDITSHNEDYTIVEQYERAEGRHSTGGMDELYKRSRSAAAGAGGAARAMKMDKKTIVWFRRDLRIEDNPALAAAAHEGSVFPVFIWCPEEEGQFYPGRASRWWMKQSLAHLSQSLKALGSDLTLIKTHNTISAILDCIRVTGATKVVFNHLYDPVSLVRDHTVKEKLVERGISVQSYNGDLLYEPWEIYCEKGKPFTSFNSYWKKCLDMSIESVMLPPPWRLMPITAAAEAIWACSIEELGLENEAEKPSNALLTRAWSPGWSNADKLLNEFIEKQLIDYAKNSKKVVGNSTSLLSPYLHFGEISVRHVFQCARMKQIIWARDKNSEGEESADLFLRGIGLREYSRYICFNFPFTHEQSLLSHLRFFPWDADVDKFKAWRQGRTGYPLVDAGMRELWATGWMHNRIRVIVSSFAVKFLLLPWKWGMKYFWDTLLDADLECDILGWQYISGSIPDGHELDRLDNPALQGAKYDPEGEYIRQWLPELARLPTEWIHHPWDAPLTVLKASGVELGTNYAKPIVDIDTARELLAKAISRTREAQIMIGAAARGAAAGAGGAGRGGGGSGSGSAGGSAGGSAGGSAGGSAGGSAGGSAGGSRGEIAKSLKEIAKSLKEIAWSLKEIAKSLKG |
| pLenti-2×P4M-mGFP  -CIBN-HOTag6-P2A-CRY2-HOTag3 | P4M: deep yellow linker: red mGFP: green CIBN: black  linker-HOTag6: blue P2A: purple CRY2:deep blue  linker-HOTag3: yellow  MTASTENFKNVKEKYQQMRGDALKTEILADFKDKLAEATDEQSLKQIVAELKSKDEYRILAKGQGLTTQLLGLKTSSVSSFEKMVEETRESIKSQERQTIKIKGGGSGGGSTASTENFKNVKEKYQQMRGDALKTEILADFKDKLAEATDEQSLKQIVAELKSKDEYRILAKGQGLTTQLLGLKTSSVSSFEKMVEETRESIKSQERQTIKIKGPGSGSGSMVSKGEELFTGVVPILVELDGDVNGHKFSVSGEGEGDATYGKLTLKFICTTGKLPVPWPTLVTTLTYGVQCFARYPDHMKQHDFFKSAMPEGYVQERTIFFKDDGNYKTRAEVKFEGDTLVNRIELKGIDFKEDGNILGHKLEYNYNSHKVYITADKQKNGIKVNFKTRHNIEDGSVQLADHYQQNTPIGDGPVLLPDNHYLSTQSKLSKDPNEKRDHMVLLEFVTAAGITLGMDELYASGSGSMNGAIGGDLLLNFPDMSVLERQRAHLKYLNPTFDSPLAGFFADSSMITGGEMDSYLSTAGLNLPMMYGETTVEGDSRLSISPETTLGTGNFKAAKFDTETKDCNEAAKKMTMNRDDLVEEGEEEKSKITEQNNGSTKSIKKMKHKAKKEENNFSNDSSKVTKELEKTDYIHVGSGSAGGSAGGSAGGSAGGSAGGSAGGSAGGSRTLREIEELLRKIIEDSVRSVAELEDIEKWLKKIGSGATNFSLLKQAGDVEENPGPMKMDKKTIVWFRRDLRIEDNPALAAAAHEGSVFPVFIWCPEEEGQFYPGRASRWWMKQSLAHLSQSLKALGSDLTLIKTHNTISAILDCIRVTGATKVVFNHLYDPVSLVRDHTVKEKLVERGISVQSYNGDLLYEPWEIYCEKGKPFTSFNSYWKKCLDMSIESVMLPPPWRLMPITAAAEAIWACSIEELGLENEAEKPSNALLTRAWSPGWSNADKLLNEFIEKQLIDYAKNSKKVVGNSTSLLSPYLHFGEISVRHVFQCARMKQIIWARDKNSEGEESADLFLRGIGLREYSRYICFNFPFTHEQSLLSHLRFFPWDADVDKFKAWRQGRTGYPLVDAGMRELWATGWMHNRIRVIVSSFAVKFLLLPWKWGMKYFWDTLLDADLECDILGWQYISGSIPDGHELDRLDNPALQGAKYDPEGEYIRQWLPELARLPTEWIHHPWDAPLTVLKASGVELGTNYAKPIVDIDTARELLAKAISRTREAQIMIGAAARGAAAGAGGAGRGGGGSGSGSAGGSAGGSAGGSAGGSAGGSAGGSAGGSRGEIAKSLKEIAKSLKEIAWSLKEIAKSLKG |
| pLenti-NES-2×PABP-mGFP-CIBN-HOTag6-P2A-CRY2-HOTag3 | NES-2×PABP:deep yellow linker: red mGFP:green CIBN: black  linker-HOTag6: blue P2A: purple CRY2:deep blue  linker-HOTag3: yellow  MSRANSNELALKLAGLDINKTESRMDNCSASRRRDRLHVKLKSLRNKIHKQLHPNCRFDDATKTSGGGSGGGSMDNCSGSRRRDRLHVKLKSLRNKIHKQLHPNCRFDDATKTSGPGSGSGSMVSKGEELFTGVVPILVELDGDVNGHKFSVSGEGEGDATYGKLTLKFICTTGKLPVPWPTLVTTLTYGVQCFARYPDHMKQHDFFKSAMPEGYVQERTIFFKDDGNYKTRAEVKFEGDTLVNRIELKGIDFKEDGNILGHKLEYNYNSHKVYITADKQKNGIKVNFKTRHNIEDGSVQLADHYQQNTPIGDGPVLLPDNHYLSTQSKLSKDPNEKRDHMVLLEFVTAAGITLGMDELYASGSGSMNGAIGGDLLLNFPDMSVLERQRAHLKYLNPTFDSPLAGFFADSSMITGGEMDSYLSTAGLNLPMMYGETTVEGDSRLSISPETTLGTGNFKAAKFDTETKDCNEAAKKMTMNRDDLVEEGEEEKSKITEQNNGSTKSIKKMKHKAKKEENNFSNDSSKVTKELEKTDYIHVGSGSAGGSAGGSAGGSAGGSAGGSAGGSAGGSRTLREIEELLRKIIEDSVRSVAELEDIEKWLKKIGSGATNFSLLKQAGDVEENPGPMKMDKKTIVWFRRDLRIEDNPALAAAAHEGSVFPVFIWCPEEEGQFYPGRASRWWMKQSLAHLSQSLKALGSDLTLIKTHNTISAILDCIRVTGATKVVFNHLYDPVSLVRDHTVKEKLVERGISVQSYNGDLLYEPWEIYCEKGKPFTSFNSYWKKCLDMSIESVMLPPPWRLMPITAAAEAIWACSIEELGLENEAEKPSNALLTRAWSPGWSNADKLLNEFIEKQLIDYAKNSKKVVGNSTSLLSPYLHFGEISVRHVFQCARMKQIIWARDKNSEGEESADLFLRGIGLREYSRYICFNFPFTHEQSLLSHLRFFPWDADVDKFKAWRQGRTGYPLVDAGMRELWATGWMHNRIRVIVSSFAVKFLLLPWKWGMKYFWDTLLDADLECDILGWQYISGSIPDGHELDRLDNPALQGAKYDPEGEYIRQWLPELARLPTEWIHHPWDAPLTVLKASGVELGTNYAKPIVDIDTARELLAKAISRTREAQIMIGAAARGAAAGAGGAGRGGGGSGSGSAGGSAGGSAGGSAGGSAGGSAGGSAGGSRGEIAKSLKEIAKSLKEIAWSLKEIAKSLKG |
| pLenti-CMV-cpLOV2-NLS-mGFP-NES2(L  5A+I14A)-2×PABD(Opi1p) | cpLOV2:yellow  linker:red NES1:gray mGFP:green  NES2(L5A and I14A):gray Opi1p:deep yellow NLS:blue  MTEHVRDAAEREGVMLIKKTAENIDEAAKELAGLDLGGGSGGSGGGLATTLERIEKNFVITDPRLPDNPIIFASDSFLQLTEYSREEILGRNCRFLQGPETDRATVRKIRDAIDNQTEVTVQLINYTKSGKKFWNLFHLQPMRDQKGDVQYFIGVQLDGGSGSGSAAAKRSWSMAFGSGSGSMVSKGEELFTGVVPILVELDGDVNGHKFSVSGEGEGDATYGKLTLKFICTTGKLPVPWPTLVTTLTYGVQCFSRYPDHMKQHDFFKSAMPEGYVERTIFFKDDGNYKTRAEVKFEGDTLVNRIELKGIDFKEDGNILGHKLEYNYNSHNVYIMADKQKNGIKVNFKIRHNIEDGSVQLADHYQQNTPIGDGPVLLPDNHYLSTQSKLSKDPNEKRDHMVLLEFVTAAGITLGMDELYKSGLRSRANSNE**A**ALKLAGLD**A**NKTESRGSGSGSKRQKLSRAIAKGKDNLKEYKLNMSIESKKRLVTCLHLLKLANKQLSDKISCLQDLVEKEQGSGSKRQKLSRAIAKGKDNLKEYKLNMSIESKKRLVTCLHLLKLANKQLSDKISCLQDLVEKEQ |
| pLenti-CMV-cpLOV2-NLS-mGFP-NES2(L5A+I14A)-3×PABD(Raf1) | cpLOV2:yellow linker:red NES1:gray mGFP:green  NES2(L5A and I14A):gray Raf1:deep yellow NLS:blue  MTEHVRDAAEREGVMLIKKTAENIDEAAKELAGLDLGGGSGGSGGGLATTLERIEKNFVITDPRLPDNPIIFASDSFLQLTEYSREEILGRNCRFLQGPETDRATVRKIRDAIDNQTEVTVQLINYTKSGKKFWNLFHLQPMRDQKGDVQYFIGVQLDGGSGSGSAAAKRSWSMAFGSGSGSMVSKGEELFTGVVPILVELDGDVNGHKFSVSGEGEGDATYGKLTLKFICTTGKLPVPWPTLVTTLTYGVQCFSRYPDHMKQHDFFKSAMPEGYVQERTIFFKDDGNYKTRAEVKFEGDTLVNRIELKGIDFKEDGNILGHKLEYNYNSHNVYIMADKQKNGIKVNFKIRHNIEDGSVQLADHYQQNTPIGDGPVLLPDNHYLSTQSKLSKDPNEKRDHMVLLEFVTAAGITLGMDELYKSGLRSRANSNE**A**ALKLAGLD**A**NKTESRPEQFQAFRNEVAVLRKTRHVNILLFMGYMTKDNLAIVTQWCEGGGGSGGGSPEQFQAFRNEVAVLRKTRHVNILLFMGYMTKDNLAIVTQWCEGGGGSGGGSPEQFQAFRNEVAVLRKTRHVNILLFMGYMTKDNLAIVTQWCEG |
| pLenti-CMV-cpLOV2  -NLS-mGFP-NES2(L5A+I14A)-2×PABD(4E mutations) | cpLOV2:yellow linker:red NES1:gray mCherry:shiny red  NES2(L5A+I14A):gray PABD(4E mutation):deep yellow NLS:blue  MTEHVRDAAEREGVMLIKKTAENIDEAAKELAGLDLGGGSGGSGGGLATTLERIEKNFVITDPRLPDNPIIFASDSFLQLTEYSREEILGRNCRFLQGPETDRATVRKIRDAIDNQTEVTVQLINYTKSGKKFWNLFHLQPMRDQKGDVQYFIGVQLDGGSGSGSAAAKRSWSMAFGSGSGSMVSKGEEDNMAIIKEFMRFKVHMEGSVNGHEFEIEGEGEGRPYEGTQTAKLKVTKGGPLPFAWDILSPQFMYGSKAYVKHPADIPDYLKLSFPEGFKWERVMNFEDGGVVTVTQDSSLQDGEFIYKVKLRGTNFPSDGPVMQKKTMGWEASSERMYPEDGALKGEIKQRLKLKDGGHYDAEVKTTYKAKKPVQLPGAYNVNIKLDITSHNEDYTIVEQYERAEGRHSTGGMDELYKSGLRSRANSNE**A**ALKLAGLD**A**NKTESRMDNCSASRRRDRLHVELESLENEIHKQLHPNCRFDDATKTSGGGSGGGSMDNCSASRRRDRLHVELESLENEIHKQLHPNCRFDDATKTSSWWWWZW |
| pLenti-CMV-  mCherry-PLD2 | mCherry: shiny red linker: red PLD2: blue  MVSKGEEDNMAIIKEFMRFKVHMEGSVNGHEFEIEGEGEGRPYEGTQTAKLKVTKGGPLPFAWDILSPQFMYGSKAYVKHPADIPDYLKLSFPEGFKWERVMNFEDGGVVTVTQDSSLQDGEFIYKVKLRGTNFPSDGPVMQKKTMGWEASSERMYPEDGALKGEIKQRLKLKDGGHYDAEVKTTYKAKKPVQLPGAYNVNIKLDITSHNEDYTIVEQYERAEGRHSTGGMDELYKGGGSGGGSGSMTATPESLFPTGDELDSSQLQMESDEVDTLKEGEDPADRMHPFLAIYELQSLKVHPLVFAPGVPVTAQVVGTERYTSGSKVGTCTLYSVRLTHGDFSWTTKKKYRHFQELHRDLLRHKVLMSLLPLARFAVAYSPARDAGNREMPSLPRAGPEGSTRHAASKQKYLENYLNRLLTMSFYRNYHAMTEFLEVSQLSFIPDLGRKGLEGMIRKRSGGHRVPGLTCCGRDQVCYRWSKRWLVVKDSFLLYMCLETGAISFVQLFDPGFEVQVGKRSTEARHGVRIDTSHRSLILKCSSYRQARWWAQEITELAQGPGRDFLQLHRHDSYAPPRPGTLARWFVNGAGYFAAVADAILRAQEEIFITDWWLSPEVYLKRPAHSDDWRLDIMLKRKAEEGVRVSILLFKEVELALGINSGYSKRALMLLHPNIKVMRHPDQVTLWAHHEKLLVVDQVVAFLGGLDLAYGRWDDLHYRLTDLGDSSESAASQPPTPRPDSPATPDLSHNQFFWLGKDYSNLITKDWVQLDRPFEDFIDRETTPRMPWRDVGVVVHGLPARDLARHFIQRWNFTKTTKAKYKTPTYPYLLPKSTSTANQLPFTLPGGQCTTVQVLRSVDRWSAGTLENSILNAYLHTIRESQHFLYIENQFFISCSDGRTVLNKVGDEIVDRILKAHKQGWCYRVYVLLPLLPGFEGDISTGGGNSIQAILHFTYRTLCRGEYSILHRLKAAMGTAWRDYISICGLRTHGELGGHPVSELIYIHSKVLIADDRTVIIGSANINDRSLLGKRDSELAVLIEDTETEPSLMNGAEYQAGRFALSLRKHCFGVILGANTRPDLDLRDPICDDFFQLWQDMAESNANIYEQIFRCLPSNATRSLRTLREYVAVEPLATVSPPLARSELTQVQGHLVHFPLKFLEDESLLPPLGSKEGMIPLEVWT |
| pLVX-TR3GS-Tom20-mGFP (or none)-PLD  _PMF100×_-hPGK-TetOne | Tom20:purple mGFP:green PLD_PMF100×_:orange  linker:red  MVGRNSAIAAGVCGALFIGYCIYFDRKRRSDPNFKNRLRERRKKQKLAKERAGLSKLPDLKDAEAVQKFFLEEIQLGEELLAQGEYEKGVDHLTNAIAVCGQPQQLLQVLQQTLPPPVFQMLLTKLPTISQRIVSAQSLAEDDVEGGSGDPPVATMVSKGEELFTGVVPILVELDGDVNGHKFSVSGEGEGDATYGKLTLKFICTTGKLPVPWPTLVTTLTYGVQCFARYPDHMKQHDFFKSAMPEGYVQERTIFFKDDGNYKTRAEVKFEGDTLVNRIELKGIDFKEDGNILGHKLEYNYNSHKVYITADKQKNGIKVNFKTRHNIEDGSVQLADHYQQNTPIGDGPVLLPDNHYLSTQSKLSKDPNEKRDHMVLLEFVTAAGITLGMDELYARGAAAGAGGAGRGGGGSADSATPHLDAVEQTLRQVSPGLEGDVWERTSGNKLDGSAADPSDWLLQTPGCWGDDRCVDRVGTKRLLAKMTENIGNATRTVDISTLAPFPNGAFQDAIVAGLKESAARGNKLKVRILVGAAPVYHMNVIPSKYRDELTAKLGKAAENITLNVASMTTSKTAFSWNHSKILVVDGQSALTGGINSWKDDYLDTTHPVSDVDLALTGPAAGSAGRYLDTLWTWTCQNKSNIASVWFAASGNAGCMATMHKDTNPKASPATGNVPIIAVGGLGVGIKDVDPKSTFRPDLPTASDTKCVVGLHDNTNADRDYDTVNPEESALRALVASAKSHIEISQQDLNATCPPLPRYDIRLYDALAAKMAAGVKVRIVVSDPANRGAVGSVGYSQIKSLSEISDTLRNRLANITGSQQAAKTAMCSNLQLATFRSSPNDKWADGHPYAQHHKLVSVDSSTFYIGSKNLYPSWLQDFGYIVESPEAAKQLDAKLLDPQWKYSQETATVDYARGICNAEF |
| pLVX-TR3GS-  miRFP670nano3-  24×GCN4-NES2(L5A+I14A)-2×PABD-  hPGK-TetOne | miRFP670nano3:grey violet 24×GCN4:pink NES2(L5A and I14A):gray 2×PABP:deep yellow linker:red  MANLDKMLNTTVTEVRKFLQADRVCVFKFEEDYSGTVSHEAVDDRWISILKTQVQDRYFMETRGEEYVHGRYQAIADIYTANLVECYRDLLIEFQVRAILAVPILQGKKLWGLLVAHQLAGPREWQTWEIDFLKQQAVVMGIAIQQSATMVGPGGSGGGGSGGEELLSKNYHLENEVARLKKGSGSGEELLSKNYHLENEVARLKKGSGSGEELLSKNYHLENEVARLKKGSGSGEELLSKNYHLENEVARLKKGSGSGEELLSKNYHLENEVARLKKGSGSGEELLSKNYHLENEVARLKKGSGSGEELLSKNYHLENEVARLKKGSGSGEELLSKNYHLENEVARLKKGSGSGEELLSKNYHLENEVARLKKGSGSGEELLSKNYHLENEVARLKKGSGSGEELLSKNYHLENEVARLKKGSGSGEELLSKNYHLENEVARLKKGSGSGEELLSKNYHLENEVARLKKGSGSGEELLSKNYHLENEVARLKKGSGSGEELLSKNYHLENEVARLKKGSGSGEELLSKNYHLENEVARLKKGSGSGEELLSKNYHLENEVARLKKGSGSGEELLSKNYHLENEVARLKKGSGSGEELLSKNYHLENEVARLKKGSGSGEELLSKNYHLENEVARLKKGSGSGEELLSKNYHLENEVARLKKGSGSGEELLSKNYHLENEVARLKKGSGSGEELLSKDYHLENEVARLKKGSGSGEELLSKNYHLENEVARLKKGSGSGSGLRSRANSNE**A**ALKLAGLD**A**NKTESRMDNCSGSRRRDRLHVKLKSLRNKIHKQLHPNCRFDDATKTSGGGSGGGSMDNCSGSRRRDRLHVKLKSLRNKIHKQLHPNCRFDDATKTS |
| pLenti-CMV-mCh-ARL11-P2A-Tom20-miRFP670nano3-PLD1 | mCherry: deep red linker:red ARL11:green P2A:blue  Tom20:purple miRFP670nano3:grey violet PLD1:acid blue  MVSKGEEDNMAIIKEFMRFKVHMEGSVNGHEFEIEGEGEGRPYEGTQTAKLKVTKGGPLPFAWDILSPQFMYGSKAYVKHPADIPDYLKLSFPEGFKWERVMNFEDGGVVTVTQDSSLQDGEFIYKVKLRGTNFPSDGPVMQKKTMGWEASSERMYPEDGALKGEIKQRLKLKDGGHYDAEVKTTYKAKKPVQLPGAYNVNIKLDITSHNEDYTIVEQYERAEGRHSTGGMDELYKGSGSATMGSVNSRGHKAEAQVVMMGLDSAGKTTLLYKLKGHQLVETLPTVGFNVEPLKAPGHVSLTLWDVGGQAPLRASWKDYLEGTDILVYVLDSTDEARLPESAAELTEVLNDPNMAGVPFLVLANKQEAPDALPLLKIRNRLSLERFQDHCWELRGCSALTGEGLPEALQSLWSLLKSRSCMCLQARAHGAERGDSKRSGSGSATNFSLLKQAGDVEENPGPMVGRNSAIAAGVCGALFIGYCIYFDRKRRSDPNFKNRLRERRKKQKLAKERAGLSKLPDLKDAEAVQKFFLEEIQLGEELLAQGEYEKGVDHLTNAIAVCGQPQQLLQVLQQTLPPPVFQMLLTKLPTISQRIVSAQSLAEDDVEGGSGDPPVATGSGSMANLDKMLNTTVTEVRKFLQADRVCVFKFEEDYSGTVSHEAVDDRWISILKTQVQDRYFMETRGEEYVHGRYQAIADIYTANLVECYRDLLIEFQVRAILAVPILQGKKLWGLLVAHQLAGPREWQTWEIDFLKQQAVVMGIAIQQSGSGSGNSYRHARWWGGAIEEFIQKHGTNFLKDHRFGSYAAIQENALAKWYVNAKGYFEDVANAMEEANEEIFITDWWLSPEIFLKRPVVEGNRWRLDCILKRKAQQGVRIFIMLYKEVELALGINSEYTKRTLMRLHPNIKVMRHPDHVSSTVYLWAHHEKLVIIDQSVAFVGGIDLAYGRWDDNEHRLTDVGSVKRVTSGPSLGSLPPAAMESMESLRLKDKNEPVQNLPIQKSIDDVDSKLKGIGKPRKFSKFSLYKQLHRHHLHDADSISSIDSTSNTGSIRSLQTGVGELHGETRFWHGKDYCNFVFKDWVQLDKPFADFIDRYSTPRMPWHDIASAVHGKAARDVARHFIQRWNFTKIMKSKYRSLSYPFLLPKSQTTAHELRYQVPGSVHANVQLLRSAADWSAGIKYHEESIHAAYVHVIENSRHYIYIENQFFISCADDKVVFNKIGDAIAQRILKAHRENQKYRVYVVIPLLPGFEGDISTGGGNALQAIMHFNYRTMCRGENSILGQLKAELGNQWINYISFCGLRTHAELEGNLVTELIYVHSKLLIADDNTVIIGSANINDRSMLGKRDSEMAVIVQDTETVPSVMDGKEYQAGRFARGLRLQCFRVVLGYLDDPSEDIQDPVSDKFFKEVWVSTAARNATIYDKVFRCLPNDEVHNLIQLRDFINKPVLAKEDPIRAEEELKKIRGFLVQFPFYFLSEESLLPSVGTKEAIVPMEVWT |
